# Red-Shift (2-Hydroxyphenyl)-Benzothiazole Emission by Mimicking the Excited-State Intramolecular Proton Transfer Effect

**DOI:** 10.3389/fchem.2021.807433

**Published:** 2021-12-24

**Authors:** Yong Ren, Lei Zhou, Xin Li

**Affiliations:** ^1^ College of Pharmaceutical Sciences, Zhejiang University, Hangzhou, China; ^2^ Collaborative Innovation Center of Yangtze River Delta Region Green Pharmaceuticals, Zhejiang University of Technology, Hangzhou, China

**Keywords:** fluorescence imaging, fluorophore, benzothiazole, excited-state intramolecular proton transfer, tautomerization, wavelength, quantum yield

## Abstract

Novel strategies to optimize the photophysical properties of organic fluorophores are of great significance to the design of imaging probes to interrogate biology. While the 2-(2-hydroxyphenyl)-benzothiazole (HBT) fluorophore has attracted considerable attention in the field of fluorescence imaging, its short emission in the blue region and low quantum yield restrict its wide application. Herein, by mimicking the excited-state intramolecular proton transfer (ESIPT) effect, we designed a series of 2-(2-hydroxyphenyl)-benzothiazole (HBT) derivatives by complexing the heteroatoms therein with a boron atom to enhance the chance of the tautomerized keto-like resonance form. This strategy significantly red-shifted the emission wavelengths of HBT, greatly enhanced its quantum yields, and caused little effect on molecular size. Typically, compounds **12B** and **13B** were observed to emit in the near-infrared region, making them among the smallest organic structures with emission above 650 nm.

## Introduction

Novel design of new fluorophores has attracted increasing attention due to their fascinating applications in fields such as fluorescence imaging (Kim and Cho 2015; Fernández and Vendrell, 2016; Singh et al., 2019; Tian et al., 2021), fluorescence diagnosis (Vahrmeijer et al., 2013; Goryaynov et al., 2019; Hernot et al., 2019), or as light-emitting diodes (LEDs) (Yang et al., 2017; Liu et al., 2018). In particular, organic small-molecule fluorophores are extremely popular due to their well-defined structures, easy synthesis, and consistency between batches. For an organic small-molecule fluorophore, its chemical structure determines its fluorescence properties including fluorescence excitation wavelength, emission wavelength, Stokes shift and quantum yield. And these fluorescence properties greatly determine the application scope of the fluorophore in various research fields. Under this circumstance, the design of new small-molecule fluorophores with desirable properties has attracted much attention (Yuan et al., 2012; Cheng et al., 2016; Qian et al., 2017; Xiang et al., 2019). Generally, there are two strategies widely used for new fluorophore development; one is *de novo* design (Xiang et al., 2019), and the other is structure optimization of classical fluorophores such as BODIPY (Ulrich et al., 2008; Boens et al., 2012; Lu et al., 2014; Wang et al., 2020), rhodamine (Lefevre et al., 1996; Fu et al., 2008; Song et al., 2008; Koide et al., 2012; Grimm et al., 2015; Chu et al., 2016; Liu et al., 2016a; Grimm et al., 2017; Lei et al., 2017; Grimm et al., 2020), cyanine (Michie et al., 2017; Usama et al., 2019; Li et al., 2020a). The latter strategy is especially appealing because the classical fluorophores usually show good photophysical properties which set a good starting point for further structure modification.

2-(2-Hydroxyphenyl)-benzothiazole (HBT) is a well-recognized fluorophore with unique properties such as good photostability and solid-based emission. This fluorophore has attracted dramatic interest in the field of bio-imaging (Li et al., 2015; Barman et al., 2016; Li et al., 2020b). However, there are several disadvantages regarding this fluorophore, e.g., its short emission wavelength and low quantum yield. The development of HBT derivatives with longer emission wavelength and improved quantum yields is highly desirable. Herein, we reported the design and synthesis of a new series of HBT derivatives, among which, compounds **12B** and **13B** demonstrated not only near-infrared emission, but significantly improved brightness compared to HBT. Their applicability for live cell imaging was also confirmed. These improved features demonstrate the feasibility of our design strategy, and potentiate the wider application scope for HBT and its derivatives.

## Results

### Design and Synthesis of 2-(2-Hydroxyphenyl)-Benzothiazole Derivatives Inspired by Excited-State Intramolecular Proton Transfer Effect

Traditionally, extending the conjugation system is a well-recognized strategy to red-shift compound absorption and emission. This is exemplified by the development of the Cyanine family fluorophores among which Cy3, Cy5 and Cy7 with increasing π conjugation showed gradually red-shifted absorption and emission. However, this often significantly increases molecular size and hydrophobicity, leading to poor solubility for aqueous-solution based applications such as bioimaging. To red shift the emission of HBT without significantly altering its other physicochemical properties, novel design strategies should be developed.

**FIGURE 1:**
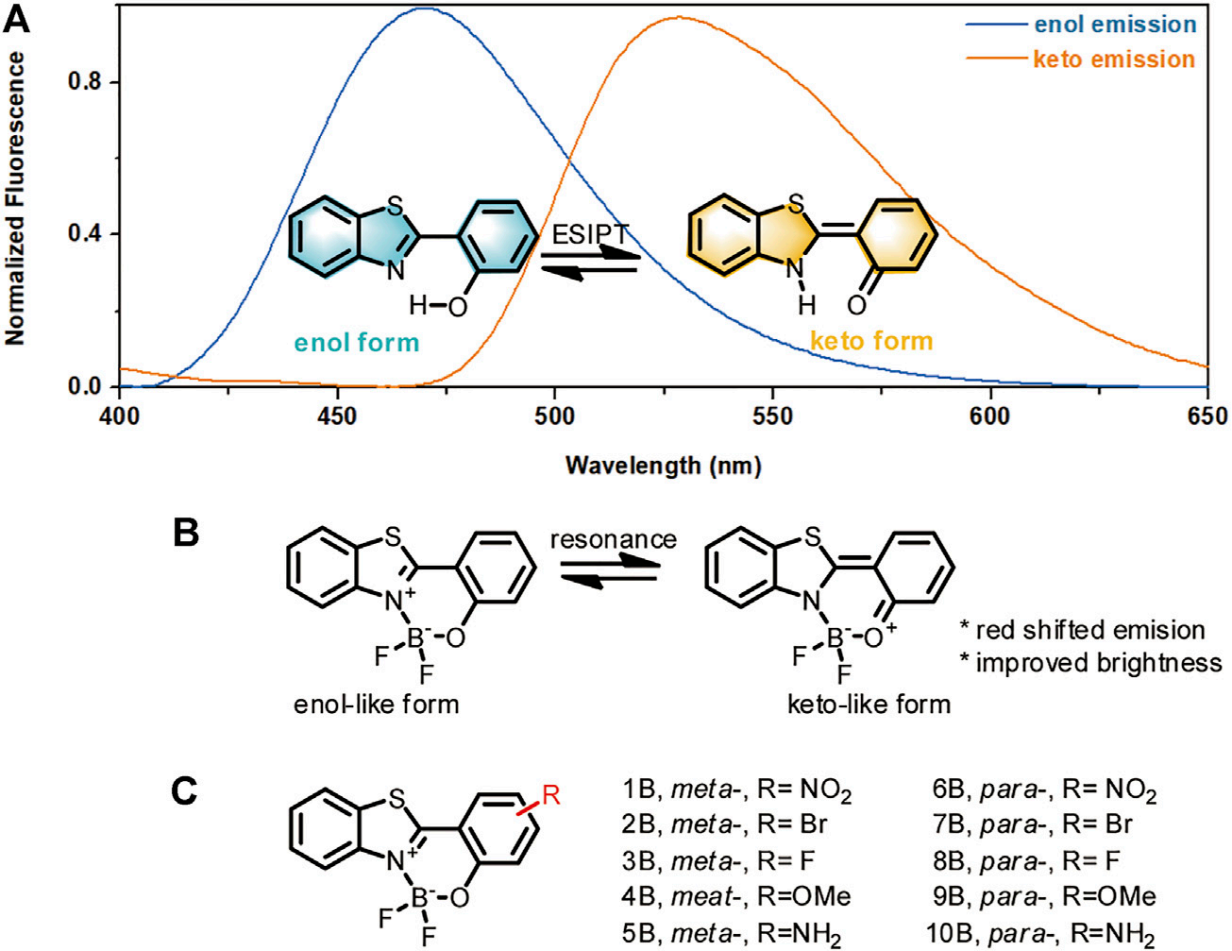
Design rationale for HBT-BF_2_ fluorophores. **(A)** Enol and keto forms of HBT structures and their emissions. **(B)** The HBT-BF_2_ structure and its resonance to a keto-like structure. **(C)** Structures of HBT-BF_2_ series of fluorophores with various substituents.

It is widely known that HBT may emit in the blue light region *via* its phenolate (enol) form, or in the green-yellow light region *via* its proton-transferred (keto) form (Figure 1A). The dramatically red-shifted emission from the keto form is inspiring for structure optimization. However, this emission happens at little probability only in either the solid state or in aprotic environments. This is because the keto isomer is formed *via* the mechanism of excited-state intramolecular proton transfer (ESIPT) (Sedgwick et al., 2018), where the formation of an intramolecular hydrogen bonding is required to facilitate the tautomerization to the keto form. In this regard, we envisioned that if we could enhance the chance of intramolecular tautomerization and improve the stability of the keto form, consequently the red-shifted emission can be improved. Since HBT tautomerization necessitates the intramolecular hydrogen bonding between the phenol “H” and the thiazole “N,” we suspected that complexing the phenol “O” and thiazole “N” with a boron atom may mimic this intramolecular hydrogen bonding effect to improve the chance of tautomerization (Figure 1B). To tune the stability of the tautomerized keto-form isomer, substituents with various electron effects may be incorporated. With these considerations, a series of boron-based HBT derivatives were designed (Figure 1C). In these structures, boron-complexation was intended to improve the chance of tautomerization; while the substituents *meta*- or *para*- to the phenol group was employed to tune the stability of the keto-like resonance structure. Another advantage of this complexation would be to improve the quantum yields of the HBT derivatives by restricting HBT in a more rigid skeleton and therefore minimizing the excited energy decay. This effect is similar to that shows in BODIPY which also composes a boron-nitrogen complexation unite to lock the structure in a rigid form and consequently make the fluorophore showing robust brightness (Ulrich et al., 2008; Lu et al., 2014). Based on the above considerations, a series of boron-complexed HBT derivatives (**HBT-BF**_**2**_) were designed, with simple substituents of various electron withdrawing or donating effects being incorporated in the *meta-* or *para-* position to the phenol group (Figure 1C). It should be noted that although Santra et al. (2012) reported several of this kind of boron-complexed HBT derivatives, the structure-photophysical property relationship remains underexplored.

These **HBT**-**BF**_**2**_ fluorophores were facilely synthesized by first condensing various substituted salicylic acids with *O*-aminothiophenol to yield **1–10**, followed by complexation with BF_3_·Et_2_O to give **1B–10B**. Their structures were characterized by ^1^HNMR, ^13^CNMR, ^19^FNMR and MS spectroscopy. Data were detailed in the supporting information.

### Photophysical Properties of HBT-BF_2_ Derivatives

After preparation of the compounds, we first tested their photophysical properties, especially their fluorescence emission wavelength and brightness. By measuring their absorption and emission spectra in acetonitrile and ethyl acetate, we observed that BF_2_-complexation generally caused a ca. 20 nm red-shift of the HBT absorption, accompanied with decreased absorption intensity (Table 1; Supplementary Figures S1–S4; Supplementary Table S1) and significantly enhanced emission brightness. As shown in Table 1 and Supplementary Table S1, BF_2_-complexation dramatically enhanced the quantum yields of the HBT compounds. This phenomenon was especially obvious for the compounds bearing the substituents in the *meta*- position. Taking the data collected in acetonitrile for example, the quantum yield of **4** is 0.06 while that of **4B** is 0.85, which represented a more than ten-fold improvement. This observation agreed with the expectation that improving the rigidity of the structures by BF_2_ complexation should reduce the excited energy decay to improve their brightness.

**TABLE 1:** Absorption and emission properties of compounds **1–10**, **1B–10B** in CH_3_CN.

Compds	λ_abs_	ε	λ_em_	λ_ex_	Stokes shift	Φ_F_
	(nm)	(M^−1^cm^−1^)	(nm)	(nm)	(nm)	
**1**	368	14,600	602	374	228	0.05
**1B** ^a^	—	—	—	—	—	—
**2**	327	17,300	506	338	168	0.04
**2B**	327	10,300	408	358	50	0.06
**3**	334	22,700	442	387	55	0.03
**3B**	334	18,400	403	351	52	0.27
**4**	335	28,800	437	388	49	0.06
**4B**	350	9,690	407	359	48	0.85
**5**	353	21,800	399	346	53	0.10
**5B**	392	13,500	414	391	23	0.68
**6**	286	23,400	499	334	165	0.07
**6B** ^a^	—	—	—	—	—	—
**7**	340	15,200	399	342	57	0.02
**7B**	340	17,100	502	318	184	0.24
**8**	342	15,300	379	360	19	0.03
**8B**	341	5,730	412	361	51	0.22
**9**	360	13,300	401	342	59	0.06
**9B**	360	8,560	482	389	93	0.26
**10**	354	4,670	472	354	118	0.03
**10B**	365	1,210	597	437	160	0.06

a Not detected due to poor solubility.

**FIGURE 2:**
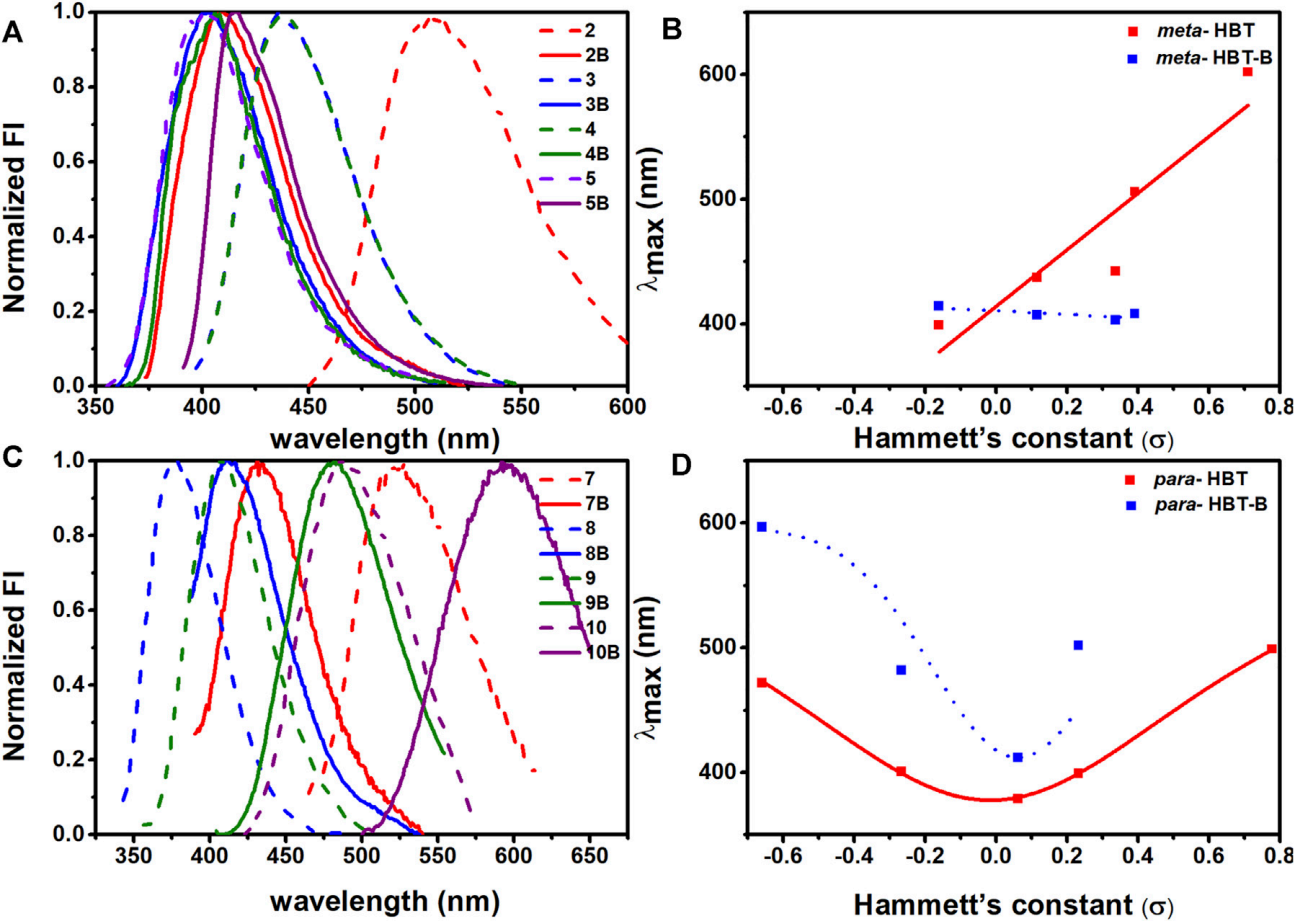
Emission spectra of **2-10**, **2B-10B** in CH_3_CN. **(A)** Normalized emission spectra of **2-5**, **2B-5B** in CH_3_CN; **(B)** Plot of the maximum emission wavelength of **2-5**, **2B-5B** against the Hammet’s constant of the R substituent. **(C)** Normalized emission spectra of **7-10**, **7B-10B** in CH_3_CN; **(D)** Plot of the maximum emission wavelength of **7-10**, **7B-10B** against the Hammett’s constant of the R substituent.

When the impact of BF_2_-complexation on emission maximum was studied, it was found that this should be discussed case-by-case (Figure 2; Supplementary Figures S5, S6). To make it clear, we plotted the maximum emission wavelength of the compounds against the Hammett’s constants of the substituents. As shown in Figures 2A,B, for the compounds bearing the substituents in the *meta-* position without BF_2_-complxation, their maximum emission wavelength increased as the substituents got more electron-withdrawing (with bigger Hammett’s constant); while BF_2_-complexation generally blue-shifted their maximum emission. For the compounds bearing the substituents in the *para-* position without BF_2_-complxation (Figures 2C,D), either the strong electron-withdrawing effect or the electron-donating effect of the substituent resulted in red-shifted emission. BF_2_-complexation generally caused a further red-shift effect. Noteworthy, compound **10** bearing a strong electron-donating amine group in the *para-* position showed its maximum emission at 472 nm; while BF_2_-complexation caused a *ca* 120 nm red shift, leading to the maximum emission at 597 nm for **10B** with part falling into the near-infrared region (>650 nm). This observation agreed with our ESIPT-mimicking design rationale, as BF_2_-complexation of the compounds bearing strong electron-donating groups in the *para-* position should make the structure more easily resonate to the keto-like structure (Figure 1C).

### Structure-Property Relationship Guided Design of HBT-BF_2_ Derivatives With Near-Infrared Emission

**FIGURE 3:**
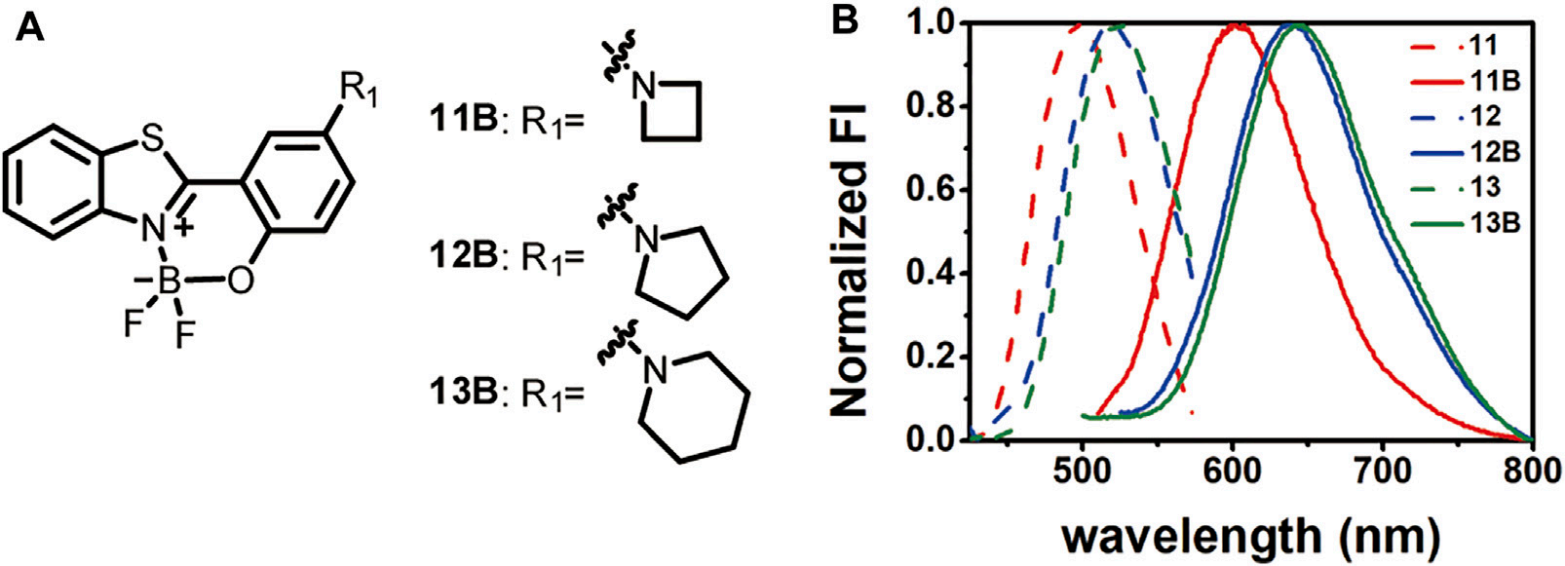
Structures of **11B-13B** **(A)** and their emission spectra in comparison to those before BF_2_-complexation in acetonitrile **(B)**.

The above work shed light on the structure-property relationship of the **HBT-BF**_**2**_ derivatives, and revealed compound **10B** as a good starting point for further optimization. To further red-shift the emission of **10B**, compounds **11–13** bearing differently sized nitrogen-containing rings were designed and synthesized, together with their BF_2_-complexed derivatives (Figure 3A). The differently sized nitrogen-containing rings were aimed to tune the twisted internal charge transfer effect, a strategy effectively used to improve the emission and brightness of Rhodamine and BODIPY (Grimm et al., 2015; Liu et al., 2016b; Grimm et al., 2017). Compounds **11–13** were synthesized by coupling **compound 7** with various amines. Subsequent complexation with BF_3_·Et_2_O yielded their BF_2_-complexed derivatives. Synthetic procedures and structure characterization data were detailed in the supporting information.

Comparison of the absorption and emission spectra of **11–13** with those of **11B–13B** showed that BF_2_-complexation resulted in a dramatic red-shifting effect, accompanied by the dramatic improvement of the fluorescence quantum yields (Figure 3B; Table 2; Supplementary Figures S7–S9). Noteworthy, **12B** and **13B** bearing a pyrrolidone and a piperidine ring, respectively, showed their emission bands centred around 650 nm in acetonitrile. Given their small size, they are among the smallest organic molecules showing NIR emission.

**TABLE 2:** Optical properties of compounds **11–13**, **11B–13B**.

Comp	λ_abs_	ε	λ_em_	λ_ex_	Stokes shift	*Φ*_F_
	(nm)	(M^−1^cm^−1^)	(nm)	(nm)	(nm)	
**11**	404	5,980	470	377	93	0.02^a^
	400	5,940	496	375	121	0.01^b^
**12**	422	5,960	482	391	91	0.02^a^
	422	5,670	517	396	121	0.01^b^
**13**	383	7,100	494	357	137	0.02^a^
	383	6,660	523	357	166	0.01^b^
**11B**	404	4,530	586	457	129	0.58^a^
	403	4,120	601	447	154	0.16^b^
**12B**	423	6,180	616	482	134	0.93^a^
	426	5,110	641	482	159	0.22^b^
**13B**	383	3,730	618	430	188	0.46^a^
	385	3,490	646	421	225	0.10^b^

a Data measured in EtOAc.

b Data measured in CH_3_CN.

**FIGURE 4:**
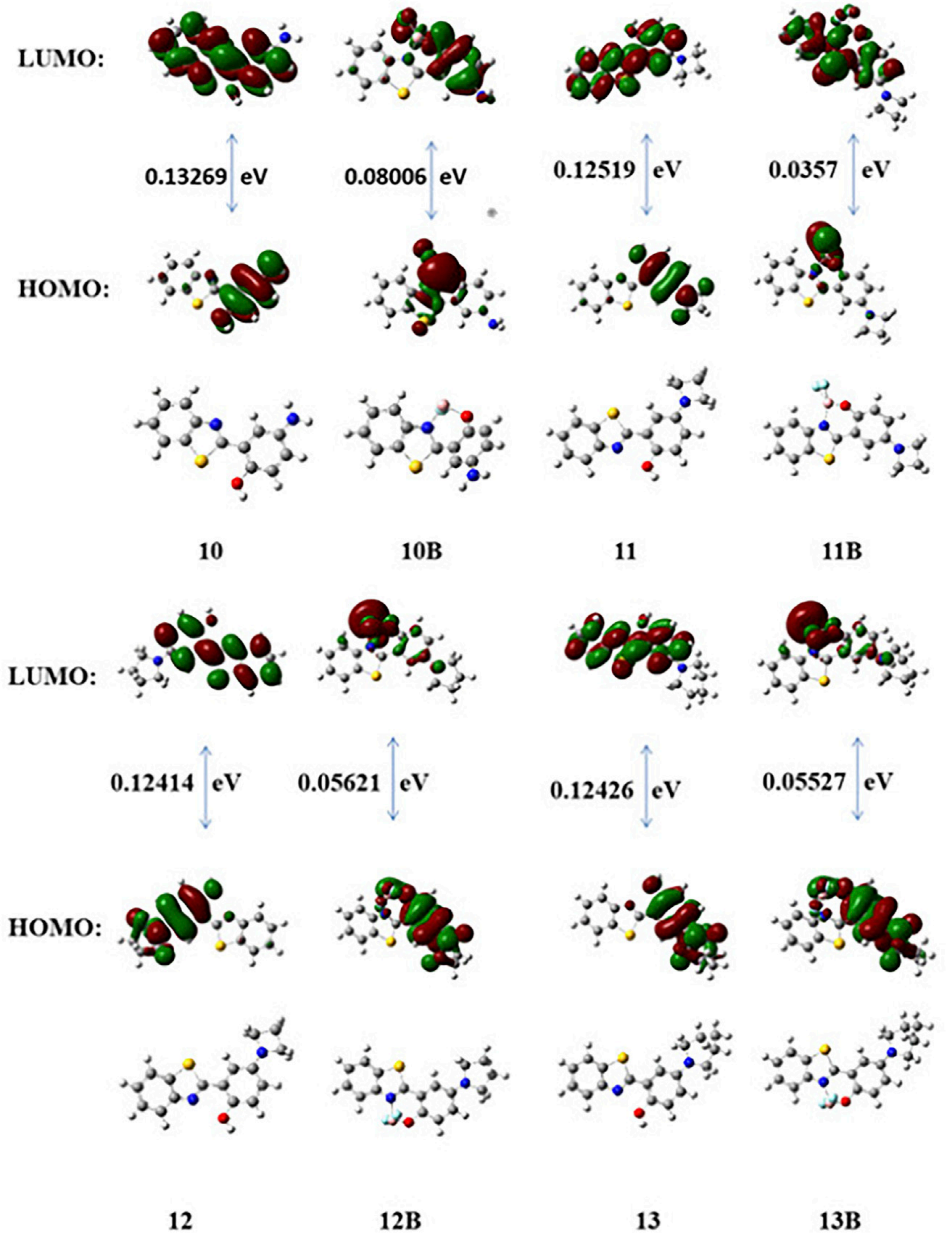
DFT optimized structures and molecular orbital plots (LUMO and HOMO) of selected fluorophores based on the optimized ground-state geometry (S_0_). In the ball-and-stick representation, carbon, nitrogen, and oxygen atoms are colored gray, blue, and red, respectively.

To further understand the relationship between the fluorescence properties and the chemical structures of these boron-based HBT derivatives, we first measured the IR spectra of **10–13** and **10B–13B** (Table 3). It was observed the BF_2_ complexation generally shifted the ν_C=N_ to higher wavenumbers, and the ν_(ph)C-N_ was also shifted a little to higher wavenumbers (Supplementary Figure S10). These results suggested that BF_2_ complexation enhanced both the electron withdrawing effect of the benzothiazole moiety and the electron donating effect of the amino substituent *para* to the phenol group when the compounds were in their ground state. Density functional theory (DFT) and time dependent density functional theory (TDDFT) calculations were also carried out with the B3LYP/6-31+G(d) method basis set using the Gaussian 09 C.01 program to further understand the structure-property relationship. The optimized geometry, the highest occupied molecular orbital (HOMO) and the lowest unoccupied molecular orbital (LUMO) of these compounds are presented in Figure 4. The results showed that complexing the heteroatoms in **10–13** with boron to lock the structures in a more rigid form (**10B–13B**) decreased their HOMO–LUMO energy gaps, which may explain their red-shifted emissions. Generally, a negative correlation can be noticed between the emission wavelength and the HOMO-LUMO energy gap for **10B–13B**, with **10B** showing the biggest energy gap demonstrating the shortest emission wavelength. Noteworthy, **12B** and **13B** demonstrated similar HOMO–LUMO energy gap values, which was in consistence with their similar emission spectra.

**TABLE 3:** Typical IR data (in cm^−1^) for compounds **11–13**, **11B–13B**.

Bond	**10**	**10B**	**11**	**11B**	**12**	**12B**	**13**	**13B**
ν_C=N_	1,499.38	1,504.1	1,493.6	1,522.2	1,504.2	1,520.6	1,509.9	1,519.1
ν_(ph)C-N_	1,441.5	1,466.5	1,426.1	1,478.1	1,431.9	1,483.9	1,436.7	1,452.1

### Applicability of 13B for Cell Imaging

**FIGURE 5:**
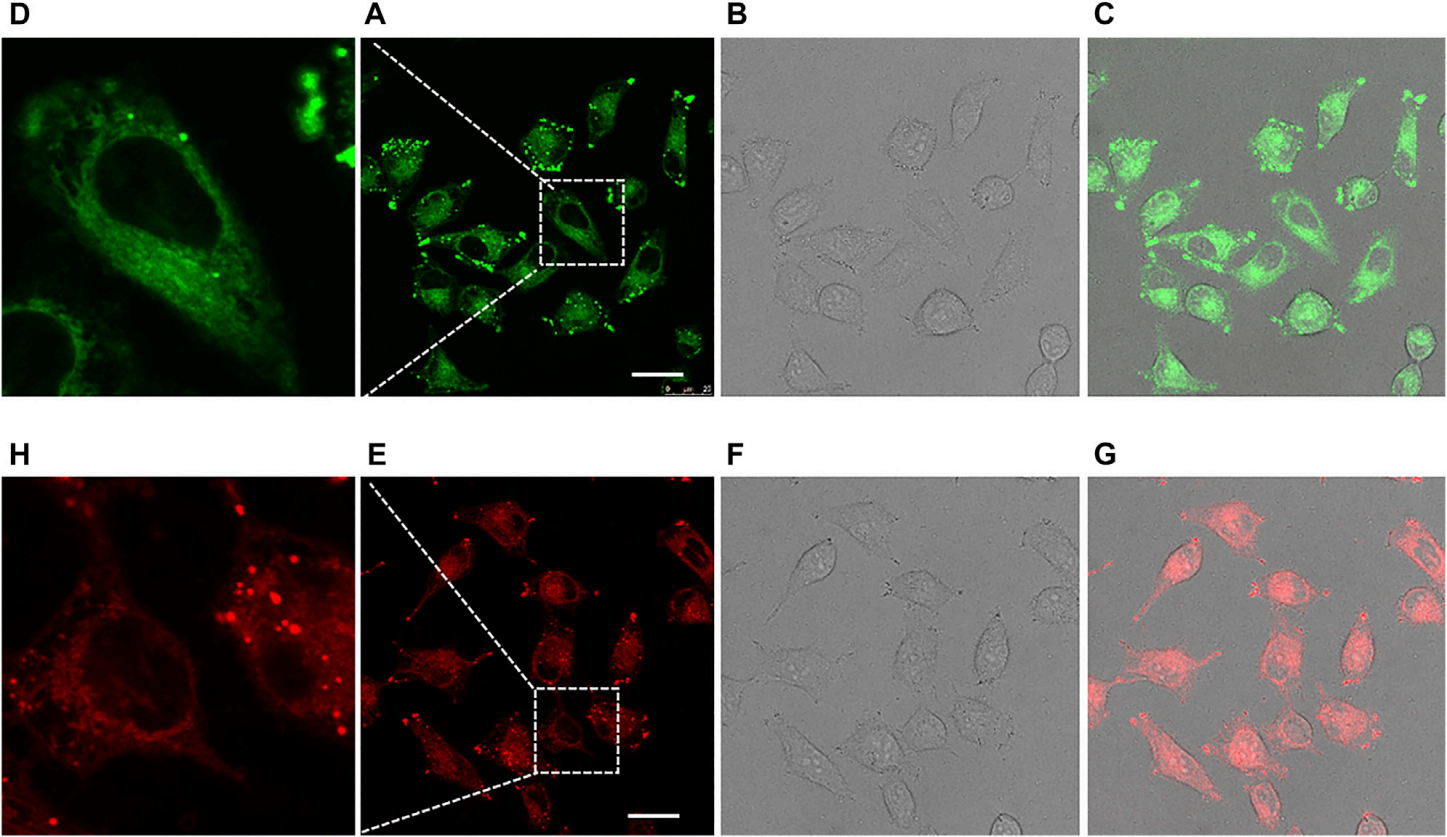
Confocal fluorescence imaging of HeLa cells cultured with **13** [Ex: 405 nm; Em: 480–600 nm; **(A–D)**] and **13B** [Ex: 405 nm; Em: 550–700 nm; **(E–H)**] for 30 min. Each compound was administrated at 20 μM. The 2nd row of pictures represented the images taken under fluorescence field, with partially expanded view shown in the 1st row. The 3rd row of pictures represented the images taken under bright field. The 4th row of pictures were merged ones. Scale bar: 25 μM.

Finally, taking compounds **13** and **13B** as representatives, we tested the feasibility of the compounds for cell imaging. For confocal fluorescence imaging, HeLa cells were first stained with 20 μM compounds **13** or **13B** at 37°C for 30 min. After that, the cells were washed three times with PBS and then imaged. As shown in Figure 5, both compounds **13** and **13B** demonstrate good cell membrane permeability and showed bright green and red light in HeLa cells, respectively. Obviously, compared with **13**, **13B** with a near infrared window emission, owned more widely applications in the fields of bioimaging as well as others.

## Discussion

Mimicking the ESIPT effect, we have developed a series of new **HBT**-based fluorophores by complexing the two key heteroatoms in HBT with a boron atom. These derivatives showed dramatically improved quantum yields. We also carried out a simple structure-property relationship study which showed that electron-donating substituents in the *para-*position to the phenol group in HBT should dramatically red-shift the emission of the **HBT-B** derivatives. Based on this observation, we developed compounds **12B** and **13B** whose relative molecular masses were below 350 but demonstrated near-infrared emission in acetonitrile, which made them among the smallest structures demonstrating near-infrared emission. These results highlighted the advantage of employing naturally occurring phenomena to design new materials with improved properties.

## Materials and Methods

### Materials and Instruments for Synthesis

All reagents for synthesis were purchased at analytical grade and used without further purification except otherwise noted. Thin-layer chromatography (TLC, 0.25 mm silica gel plates, 60 F-254, Merker KGaA) was employed to monitor the reactions and UV light was used as the visualizing agent. Flash column chromatrography over silica gel (40–63 μm particle size, Taiyang, Rushan) was employed for compound purification. ^1^H NMR and ^13^C NMR spectra were recorded on a Bruker spectrometer (500 and 126 MHz, respectively) at 23°C. NMR spectra were calibrated using TMS as the internal references. All chemical shifts were reported in parts per million (ppm) and coupling constants (*J*) in Hz. Mass spectra (HRMS) were measured on an Agilent 6224 TOF LC/MS spectrometer or a Shimadzu LCMS-2020 mass spectrometer.

### General Procedures for the Synthesis of Compounds **1–10**, **1B–10B**

A mixture of *o*-aminothiophenol, the salicylic acid derivative bearing the desired substituent, tetrabutylammonium bromide, and triphenyl phosphite was heated at 120°C for 2 h. After cooling down to ambient temperature, MeOH was added to the mixture and the mixture was sonicated. The solid was isolated by filtration and washed with MeOH, which was further purified by recrystallization to give the HBT derivative **(1–10)** ready for BF_2_-complexation.

For BF_2_-complexation, each one of compounds **1–10** was dissolved in dry dichloromethane, and was treated with triethylamine. BF_3_·Et_2_O was added dropwise to the solution at room temperature under nitrogen atmosphere. The solution was stirred at room temperature for 1 h. After the disappearance of the starting material as monitored by TLC, the solvent was directly evaporated under reduced pressure. The crude product was purified by silica gel column chromatography (eluent: PE and EA). Structure characterization data can be found in the supporting information.

### Synthesis for Compounds **11–13**, **11B–13B**

These compounds were synthesized starting with compound **7**. Compound **7** was first protected of the phenol group; then underwent coupling reaction to install the amino substitutent; subsequent deprotection yielded **10–13**; and final BF_2_-complexation gave **11B–13B**.

In detail, to a solution of compound **7** (1.0 mmol) and potassium carbonate (2.0 mmol) in dry DMF (5.0 ml) was added MOMCl (3 mmol) dropwise at 0°C. The resulting mixture was stirred at room temperature for 3 h. After the reaction was compete as monitored by TLC, the mixture was extracted with ethyl acetate. The obtained organic layer was successively washed with distilled water and brine, and was subsequently dried over anhydrous sodium sulphate. After removal of the solvent, the desired product was obtained which was used without further purification. ^1^H NMR (500 MHz, CDCl_3_) δ 8.69 (d, *J* = 2.5 Hz, 1H), 8.10 (d, *J* = 8.1 Hz, 1H), 7.92 (d, *J* = 7.9 Hz, 1H), 7.51 (ddd, *J* = 9.8, 7.9, 1.9 Hz, 2H), 7.42–7.36 (m, 1H), 7.16 (d, *J* = 8.9 Hz, 1H), 5.40 (s, 2H), 3.56 (s, 3H). ^13^C NMR (125 MHz, CDCl_3_) δ 161.31, 153.74, 151.98, 136.15, 134.12, 131.89, 126.18, 125.04, 124.59, 123.07, 121.21, 116.47, 114.89, 94.55, 56.73.

To a solution of MOM protected **7** (1.0 mmol) and the corresponding amine (2.0 mmol) in dry toluene (5.0 ml) was added cesium carbonate (3 mmol) as a base, and subsequently added Pd_2_(dba)_3_ and BINAP. Under a nitrogen atmosphere, the mixture was refluxed overnight. After the reaction was completed as monitored by TLC, the reaction mixture was filtered through Celite and the filtrate was evaporated under reduced pressure. The crude product was purified by silica gel column chromatography to yield the MOM protected **11–13** (eluent: PE and EA). These compounds were then individually dissolved in THF/H_2_O, treated with HCl (5 N) at 0°C. The mixture was stirred at 50°C overnight. After evaporation of the volatile solvent, the mixture was diluted with water and extracted with ethyl acetate. The organic phase was washed with brine, dried over anhydrous Na_2_SO_4_, and evaporated under reduced pressure. The residue was purified by silica gel column chromatography to give **11–13** (eluent: PE and EA). BF_2_-complexation was carried out as afore mentioned to give **11B–13B**. Structure characterization data can be found in the supporting information.

### General Experimental for Photophysical Property Characterization

All the photophysical characterization experiments were carried out at ambient temperature. Fluorescence measurements were acquired using an Agilent Cary Eclipse Fluorescence spectrophotometer. Absorption spectra were performed on a U-3010 Spectrophotometer.

### Quantum Yields Determination

Quantum yields were determined using quinine sulfate (Ф_standard_ = 0.577 in 0.1 M H_2_SO_4_) as astandard according to a published method. The quantum yield was calculated according to the equation:

$${Ф}_{sample}=\Phi_{s\tan dard}\cdot \frac{\;Abs_{s\tan dard}\cdot \sum F_{sample}}{\;Abs_{sample\;}\cdot \sum F_{s\tan dard}}\cdot \frac{n_{sample^{2}}}{n_{s\tan dard^{2}}}$$

where Ф is the quantum yield, Σ_F_ is the integrated fluorescence intensity, Abs is absorbance at *λ*_*ex*_ 375 nm, and *n* represents the refractive index of the solvent.

### Cell Imaging

Hela cells were cultured on glass-bottom 6-well plates overnight, then incubated with **13** or **13B** (20 μM) at 37 °C for 30 min. After three quick wash with PBS, cells were observed under a confocal microscopy (For **13**, excitation was conducted at 405 nm and emission was collected at 480–600 nm. For **13B**, excitation was conducted at 405 nm and emission was collected at 550–700 nm).

## Data Availability

The original contributions presented in the study are included in the article/Supplementary Material, further inquiries can be directed to the corresponding author.
