# Supplementary material for: Red-Shift (2-Hydroxyphenyl)-Benzothiazole Emission by Mimicking the Excited-State Intramolecular Proton Transfer Effect
Source: Front Chem. 2021 Dec 24;9:807433. doi: 10.3389/fchem.2021.807433 (PMC8738082; doi:10.3389/fchem.2021.807433)
Supplement: Supplementary file 1 [file DataSheet1.PDF]

Supplementary Material

1 Supplementary Data

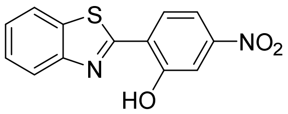

1: white powder (68% yield). ^{1}H NMR (500 MHz, $CDCl_3$) δ 8.10 – 8.05 (m, 1H), 7.97 (dd, *J* = 8.0, 0.4 Hz, 1H), 7.95 (d, *J* = 2.2 Hz, 1H), 7.85 (d, *J* = 8.6 Hz, 1H), 7.80 (dd, *J* = 8.6, 2.2 Hz, 1H), 7.62 – 7.56 (m, 1H), 7.54 – 7.48 (m, 1H). ^{13}C NMR (125 MHz, $CDCl_3$) δ 167.05, 158.41, 151.47, 149.91, 132.97, 128.98, 127.36, 126.67, 122.84, 121.76, 121.68, 114.16, 113.38. ESI-MS (**m/z**): calcd for $C_{13}H_8N_2O_3SH^+$ 273.03, found $[M+H]^+$ 273.05。

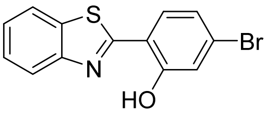

2: white powder (86% yield). ^{1}H NMR (500 MHz, $CDCl_3$) δ 7.99 – 7.96 (m, 1H), 7.90 (dd, *J* = 8.0, 0.5 Hz, 1H), 7.54 – 7.48 (m, 2H), 7.45 – 7.40 (m, 1H), 7.28 (d, *J* = 1.9 Hz, 1H), 7.08 (dd, *J* = 8.4, 1.9 Hz, 1H). ^{13}C NMR (125 MHz, $CDCl_3$) δ 168.48, 158.52, 151.59, 132.34, 129.24, 126.88, 126.46, 125.80, 122.83, 122.21, 121.56, 121.06, 115.83. ESI-MS (**m/z**): calcd for $C_{13}H_8BrNOSH^+$ 305.96, found $[M+H]^+$ 305.95.

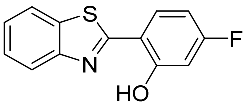

3: white powder (75% yield). ^{1}H NMR (500 MHz, $CDCl_3$) δ 7.97 (d, *J* = 8.2 Hz, 1H), 7.89 (d, *J* = 8.0 Hz, 1H), 7.65 (dd, *J* = 8.7, 6.2 Hz, 1H), 7.54 – 7.47 (m, 1H), 7.44 – 7.38 (m, 1H), 6.80 (dd, *J* = 10.4, 2.5 Hz, 1H), 6.68 (td, *J* = 8.4, 2.5 Hz, 1H). ^{13}C NMR (125 MHz, $CDCl_3$) δ 168.63, 166.02, 164.01, 159.77, 151.19, 132.02, 129.72, 126.81, 125.56, 122.07, 121.51, 107.26, 104.65. ESI-MS (**m/z**): calcd for $C_{13}H_8FNOSH^+$ 246.04, found $[M+H]^+$ 246.10.

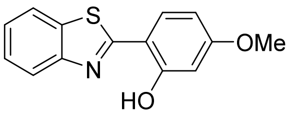

4: white powder (30% yield). ^{1}H NMR (500 MHz, $CDCl_3$) δ 7.93 (d, *J* = 8.1 Hz, 1H), 7.85 (d, *J* = 7.9 Hz, 1H), 7.57 (d, *J* = 8.7 Hz, 1H), 7.49 – 7.44 (m, 1H), 7.38 – 7.33 (m, 1H), 6.60 (d, *J* = 2.4 Hz, 1H), 6.52 (dd, *J* = 8.7, 2.4 Hz, 1H), 3.85 (s, 3H). ^{13}C NMR (125 MHz, $CDCl_3$) δ 169.27, 163.48, 159.97, 132.15, 129.64, 129.55, 126.58, 125.05, 121.64, 121.42, 110.37, 107.70, 101.34, 55.51. ESI-MS (**m/z**): calcd for $C_{14}H_{11}NO_2SH^+$ 258.06, found $[M+H]^+$ 258.08

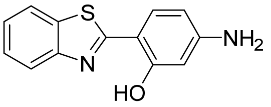

5: yellow powder (56% yield). ^{1}H NMR (500 MHz, $CDCl_3$) δ 7.88 (d, *J* = 8.0 Hz, 1H), 7.83 (d, *J* = 7.9 Hz, 1H), 7.48 – 7.40 (m, 2H), 7.37 – 7.29 (m, 1H), 6.32 (d, *J* = 2.2 Hz, 1H), 6.26 (dd, *J* = 8.4, 2.2 Hz, 1H). ^{13}C NMR (125 MHz, $CDCl_3$) δ 169.58, 159.86, 152.02, 150.99, 131.99, 130.05, 126.40, 124.63, 121.36, 121.31, 108.51, 107.22, 101.73. ESI-MS (**m/z**): calcd for $C_{13}H_{10}N_2OSH^+$ 243.06, found $[M+H]^+$ 243.10.

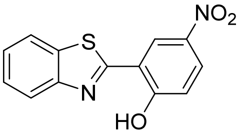

6: white powder (63% yield). ^{1}H NMR (500 MHz, $CDCl_3$) δ 8.63 (d, *J* = 2.6 Hz, 1H), 8.25 (dd, *J* = 9.1, 2.6 Hz, 1H), 8.03 (d, *J* = 8.1 Hz, 1H), 7.96 (d, *J* = 7.9 Hz, 1H), 7.60 – 7.53 (m, 1H), 7.52 – 7.44 (m, 1H), 7.18 (d, *J* = 9.1 Hz, 1H). ^{13}C NMR (125 MHz, $CDCl_3$) δ 167.31, 163.15, 151.20, 140.27, 132.65, 127.75, 127.31, 126.55, 124.51, 122.53, 121.84, 118.68, 116.45. ESI-MS (**m/z**): calcd for $C_{13}H_8N_2O_3SH^+$ 273.03, found $[M+H]^+$ 273.05.

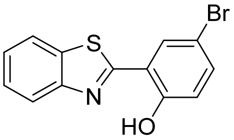

7: white powder (95% yield). ^{1}H NMR (500 MHz, $CDCl_3$) δ 7.99 (d, *J* = 8.1 Hz, 1H), 7.91 (d, *J* = 8.0 Hz, 1H), 7.77 (d, *J* = 2.2 Hz, 1H), 7.52 (t, *J* = 7.3 Hz, 1H), 7.47 – 7.37 (m, 2H), 6.99 (d, *J* = 8.8 Hz, 1H). ^{13}C NMR (125 MHz, $CDCl_3$) δ 167.74, 156.98, 151.59, 135.29, 132.58, 130.45, 126.92, 125.94, 122.35, 121.62, 119.79, 118.30, 111.01. ESI-MS (**m/z**): calcd for $C_{13}H_8BrNOSH^+$ 305.96, found $[M+H]^+$ 305.95.

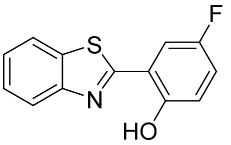

8: white powder (86% yield). ^{1}H NMR (500 MHz, $CDCl_3$) δ 7.98 (d, *J* = 8.2 Hz, 1H), 7.90 (d, *J* = 7.9 Hz, 1H), 7.54 – 7.48 (m, 1H), 7.45 – 7.40 (m, 1H), 7.36 (dd, *J* = 8.8, 2.9 Hz, 1H), 7.10 – 7.07 (m, 1H), 7.04 (dd, *J* = 9.1, 4.8 Hz, 1H). ^{13}C NMR (125 MHz, $CDCl_3$) δ 168.06, 156.55, 154.66, 154.16, 151.73, 132.66, 126.89, 125.87, 122.31, 121.58, 119.68, 119.01, 113.68. ESI-MS (**m/z**): calcd for $C_{13}H_8FNOSH^+$ 246.04, found $[M+H]^+$ 246.10.

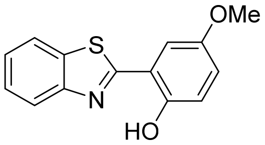

9: white powder (71% yield). ^{1}H NMR (500 MHz, $CDCl_3$) δ 8.00 (d, *J* = 8.2 Hz, 1H), 7.90 (dd, *J* = 8.0, 0.5 Hz, 1H), 7.51 (ddd, *J* = 8.3, 7.3, 1.2 Hz, 1H), 7.44 – 7.38 (m, 1H), 7.18 (d, *J* = 2.9 Hz, 1H), 7.07 – 7.02 (m, 1H), 6.99 (dd, *J* = 9.0, 2.9 Hz, 1H), 3.85 (s, 3H). ^{13}C NMR (125 MHz, $CDCl_3$) δ 171.17, 169.07, 152.48, 152.30, 132.67, 126.77, 125.60, 122.22, 121.54, 119.93, 118.77, 116.42, 111.93, 56.03. ESI-MS (**m/z**): calcd for $C_{14}H_{11}NO_2SH^+$ 258.06, found $[M+H]^+$ 258.10.

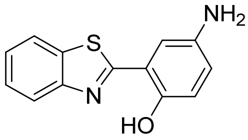

10: yellow powder (47% yield). ^{1}H NMR (500 MHz, $CDCl_3$) δ 8.66 (d, *J* = 2.6 Hz, 1H), 8.27 (dd, *J* = 9.1, 2.6 Hz, 1H), 8.05 (d, *J* = 7.9 Hz, 1H), 7.98 (dd, *J* = 8.0, 0.4 Hz, 1H), 7.61 – 7.54 (m, 1H), 7.53 – 7.47 (m, 1H), 7.20 (d, *J* = 9.1 Hz, 1H). ^{13}C NMR (125 MHz, $CDCl_3$) δ 167.34, 163.17, 151.22, 140.30, 132.67, 127.78, 127.32, 126.56, 124.55, 122.55, 121.86, 118.70, 116.49. ESI-MS (**m/z**): calcd for $C_{13}H_{10}N_2OSH^+$ 243.06, found $[M+H]^+$ 243.10.

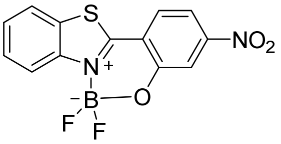

1B: yellow powder (78% yield). ^{1}H NMR (500 MHz, $CDCl_3$) δ 8.48 (d, *J* = 8.3 Hz, 1H), 8.07 – 8.00 (m, 2H), 7.87 (d, *J* = 0.7 Hz, 2H), 7.77 (t, *J* = 7.7 Hz, 1H), 7.69 (t, *J* = 7.7 Hz, 1H). ^{13}C NMR (125 MHz, $CDCl_3$) δ 129.57, 128.48, 127.93, 124.19, 122.29, 121.19, 120.26, 119.65, 119.30, 117.54, 115.89, 114.87, 113.31. ^{19}F NMR (376 MHz, CDCl3) δ (-)135.69- (-)135.74 (m, 2F). calcd for $C_{13}H_7BF_2N_2O_3SH^+$ 321.03, found $[M+H]^+$ 321.05.

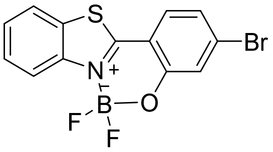

2B: white powder (50% yield). ^{1}H NMR (500 MHz, $CDCl_3$) δ 8.39 (d, *J* = 8.4 Hz, 1H), 7.93 (d, *J* = 8.1 Hz, 1H), 7.71 – 7.66 (m, 1H), 7.62 – 7.56 (m, 1H), 7.51 (d, *J* = 8.4 Hz, 1H), 7.41 (d, *J* = 1.8 Hz, 1H), 7.17 (dd, *J* = 8.4, 1.8 Hz, 1H). ^{13}C NMR (126 MHz, $CDCl_3$) δ 168.20, 156.55, 143.52, 131.53, 129.02, 127.66, 127.62, 124.14, 123.58, 122.09, 120.67, 120.65, 112.21. ^{19}F NMR (376 MHz, $CDCl_3$) δ (-)135.23-(-)135.29 (m, 2F). calcd for $C_{13}H_7BBrF_2NOSH^+$ 353.96, found $[M+H]^+$ 353.97.

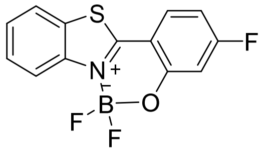

3B: white powder (91% yield). ^{1}H NMR (500 MHz, $CDCl_3$) δ 8.37 (d, *J* = 8.3 Hz, 1H), 7.94 – 7.90 (m, 1H), 7.71 – 7.64 (m, 2H), 7.58 (td, *J* = 7.8, 1.0 Hz, 1H), 6.90 (dd, *J* = 10.2, 2.4 Hz, 1H), 6.78 (ddd, *J* = 8.7, 8.0, 2.4 Hz, 1H). ^{13}C NMR (125 MHz, $CDCl_3$) δ 169.34, 167.29, 143.45, 129.04, 128.95, 128.92, 127.45, 122.05, 120.54, 120.51, 120.48, 109.57, 109.37. ^{19}F NMR (376 MHz, $CDCl_3$) δ (-)98.44-(-)98.50 (m, 1F), (-)136.82-(-)136.92 (m, 2F). calcd for $C_{13}H_7BF_3NOSH^+$ 294.04, found $[M+H]^+$ 294.08.

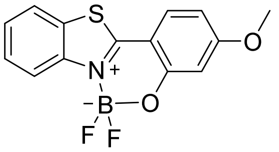

4B: white powder (42% yield). ^{1}H NMR (500 MHz, $CDCl_3$) δ 8.29 (d, *J* = 8.3 Hz, 1H), 7.86 (d, *J* = 8.1 Hz, 1H), 7.62 (ddd, *J* = 8.4, 7.4, 1.1 Hz, 1H), 7.55 – 7.48 (m, 2H), 6.64 (d, *J* = 2.4 Hz, 1H), 6.61 (dd, *J* = 8.8, 2.4 Hz, 1H). ^{13}C NMR (125 MHz, $CDCl_3$) δ 168.56, 167.28, 158.76, 143.46, 128.54, 128.35, 126.75, 121.97, 119.90, 119.88, 119.85, 110.57, 102.33. ^{19}F NMR (376 MHz, $CDCl_3$) δ (-)136.58-(-)136.68 (m, 2F). calcd for $C_{14}H_{10}BF_2NO_2SH^+$ 306.06, found $[M+H]^+$ 306.08.

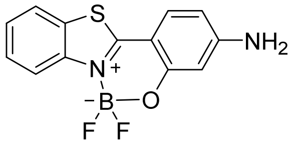

5B: yellow powder (30% yield). ^{1}H NMR (500 MHz, $CDCl_3$) δ 12.75 (dd, *J* = 21.1, 12.6 Hz, 1H), 12.64 – 12.53 (m, 2H), 12.29 – 12.20 (m, 1H), 12.13 (dd, *J* = 11.1, 7.4 Hz, 1H), 12.06 (dd, *J* = 8.7, 2.9 Hz, 1H), 11.06 (t, *J* = 6.6 Hz, 1H), 10.63 (d, *J* = 7.7 Hz, 1H). ^{13}C NMR (125 MHz, DMSO) δ 167.12, 157.46, 157.35, 142.07, 128.61, 127.26, 125.02, 122.15, 117.36, 116.69, 108.81, 101.78, 99.06. ^{19}F NMR (376 MHz, $CDCl_3$) δ (-)152.17-(-)152.22 (m, 2F). calcd for $C_{13}H_9BF_2N_2OSH^+$ 291.06, found $[M+H]^+$ 291.09.

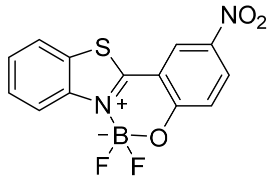

6B: yellow powder (39% yield). ^{1}H NMR (500 MHz, $CDCl_3$) δ 8.38 (s, 1H), 8.09 (d, *J* = 9.6 Hz, 1H), 7.99 (d, *J* = 7.9 Hz, 1H), 7.82 (d, *J* = 8.2 Hz, 1H), 7.42 (t, *J* = 7.8 Hz, 1H), 7.35 (t, *J* = 7.6 Hz, 1H), 6.96 (d, *J* = 9.2 Hz, 1H). ^{19}F NMR (376 MHz, $CDCl_3$) δ (-)136.09- (-)136.18 (m, 2F). calcd for $C_{13}H_7BF_2N_2O_3SH^+$ 321.03, found $[M+H]^+$ 321.05.

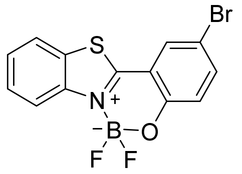

7B: yellow powder (82% yield). ^{1}H NMR (500 MHz, $CDCl_3$) δ 8.41 (d, *J* = 8.4 Hz, 1H), 7.89 (d, *J* = 8.0 Hz, 1H), 7.68 (t, *J* = 7.6 Hz, 1H), 7.56 (t, *J* = 7.6 Hz, 1H), 7.25 – 7.22 (m, 1H), 6.92 (d, *J* = 2.8 Hz, 1H), 6.78 (d, *J* = 2.7 Hz, 1H). ^{13}C NMR (125 MHz, $CDCl_3$) δ 151.65, 148.26, 143.63, 139.38, 129.48, 128.81, 128.63, 127.28, 126.39, 125.87, 122.01, 120.48, 108.84. ^{19}F NMR (376 MHz, $CDCl_3$) δ (-)136.62-(-)136.71 (m, 2F). calcd for $C_{13}H_7BBrF_2NOSH^+$ 353.96, found $[M+H]^+$ 353.98.

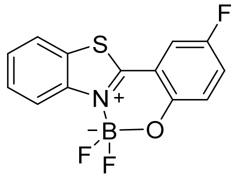

8B: yellow powder (82% yield). ^{1}H NMR (500 MHz, $CDCl_3$) δ 8.42 (d, *J* = 8.3 Hz, 1H), 7.96 (d, *J* = 8.1 Hz, 1H), 7.71 (ddd, *J* = 8.4, 7.3, 1.1 Hz, 1H), 7.63 – 7.58 (m, 1H), 7.37 – 7.30 (m, 2H), 7.20 (ddd, *J* = 8.8, 4.4, 0.6 Hz, 1H). ^{13}C NMR (125 MHz, $CDCl_3$) δ 156.72, 154.75, 152.77, 143.64, 129.09, 127.74, 124.92, 124.74, 122.15, 120.82, 111.70, 111.50, 99.86. ^{19}F NMR (376 MHz, $CDCl_3$) δ (-)122.45-(-)122.50 (m, 1F), (-)136.46-(-)136.55 (m, 2F). calcd for $C_{13}H_7BF_3NOSH^+$ 294.04, found $[M+H]^+$ 294.08.

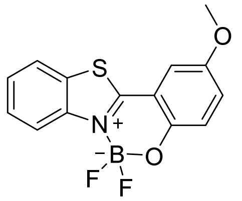

9B: yellow powder (79% yield). ^{1}H NMR (500 MHz, $CDCl_3$) δ 8.41 (d, *J* = 8.2 Hz, 1H), 7.93 – 7.91 (m, 1H), 7.68 (ddd, *J* = 8.4, 7.3, 1.2 Hz, 1H), 7.61 – 7.53 (m, 1H), 7.23 (dd, *J* = 9.1, 2.9 Hz, 1H), 7.17 (d, *J* = 9.1 Hz, 1H), 7.03 (d, *J* = 2.9 Hz, 1H). ^{13}C NMR (125 MHz, $CDCl_3$) δ 153.11, 138.16, 136.22, 134.40, 130.52, 129.72, 128.86, 127.32, 125.74, 122.01, 121.55, 120.65, 108.35, 56.13. ^{19}F NMR (376 MHz, $CDCl_3$) δ (-)136.19-(-)136.27 (m, 2F). calcd for $C_{14}H_{10}BF_2NO_2SH^+$ 306.06, found $[M+H]^+$ 306.08.

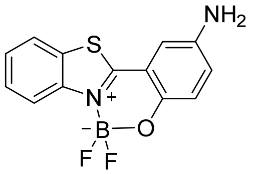

10B: yellow powder (30% yield). ^{1}H NMR (500 MHz, DMSO) δ 8.13 (d, *J* = 7.4 Hz, 1H), 8.03 (d, *J* = 8.0 Hz, 1H), 7.59 – 7.49 (m, 2H), 7.47 – 7.40 (m, 1H), 6.87 (d, *J* = 8.5 Hz, 1H), 6.83 (d, *J* = 8.5 Hz, 1H). ^{13}C NMR (125 MHz, DMSO) δ 134.69, 134.22, 133.14, 132.73, 126.91, 125.38, 122.49, 122.42, 121.52, 120.58, 118.63, 118.15, 118.11. ^{19}F NMR (376 MHz, $CDCl_3$) δ (-)136.44-(-)136.50 (m, 2F). calcd for $C_{13}H_9BF_2N_2OSH^+$ 291.06, found $[M+H]^+$ 291.09.

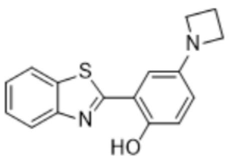

11: yellow powder (46% yield). ^{1}H NMR (500 MHz, Chloroform-*d*) δ 7.94 – 7.89 (m, 1H), 7.80 (d, *J* = 8.0 Hz, 1H), 7.46 (t, *J* = 7.4 Hz, 2H), 7.35 (t, *J* = 7.7 Hz, 1H), 7.16 (d, *J* = 8.0 Hz, 1H), 6.99 (d, *J* = 8.7 Hz, 1H), 3.75 – 3.69 (m, 2H), 1.24 (t, *J* = 7.0 Hz, 3H). ^{13}C NMR (125 MHz, $CDCl_3$) δ 168.30, 159.52, 154.60, 151.62, 132.60, 126.73, 125.68, 123.46, 122.23, 121.49, 119.11, 116.86, 99.98, 41.94, 29.40. ESI-MS (**m/z**): calcd for $C_{16}H_{14}N_2OSH^+$ 283.09, found $[M+H]^+$ 283.00.

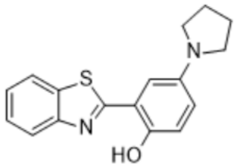

12: yellow powder (50% yield). ^{1}H NMR (500 MHz, Chloroform-*d*) δ 7.98 (dt, *J* = 8.2, 0.8 Hz, 1H), 7.89 (dt, *J* = 8.0, 0.9 Hz, 1H), 7.49 (ddd, *J* = 8.3, 7.3, 1.2 Hz, 1H), 7.39 (ddd, *J* = 8.2, 7.2, 1.2 Hz, 1H), 7.03 (d, *J* = 8.9 Hz, 1H), 6.78 (s, 2H), 3.32 (s, 4H), 2.05 (d, *J* = 3.1 Hz, 4H). ^{13}C NMR (125 MHz, $CDCl_3$) δ 169.79, 152.16, 146.58, 141.60, 134.64, 132.75, 126.58, 125.31, 122.16, 121.47, 118.50, 118.02, 116.59, 48.21, 25.39. ESI-MS (**m/z**): calcd for $C_{17}H_{16}N_2OSH^+$ 297.11, found $[M+H]^+$ 297.10.

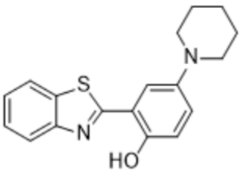

13: yellow powder (74% yield). ^{1}H NMR (500 MHz, $CDCl_3$) δ 8.09 (d, *J* = 8.1 Hz, 1H), 7.91 (d, *J* = 7.8 Hz, 1H), 7.69 (d, *J* = 3.1 Hz, 1H), 7.49 – 7.42 (m, 1H), 7.39 – 7.32 (m, 1H), 7.17 (dd, *J* = 13.0, 6.6 Hz, 1H), 6.66 (dd, *J* = 8.9, 3.1 Hz, 1H), 5.28 (s, 2H), 3.41 – 3.28 (m, 4H), 2.07 – 1.93 (m, 4H). ^{13}C NMR (125 MHz, $CDCl_3$) δ 163.75, 152.23, 146.11, 144.00, 136.27, 125.77, 124.52, 123.74,122.84, 121.15, 117.39, 115.24, 111.24, 56.45, 48.19, 25.47. ESI-MS (**m/z**): calcd for $C_{18}H_{18}N_2OSH^+$ 311.12, found $[M+H]^+$ 311.10.

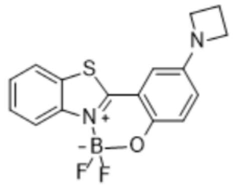

11B: red powder (33% yield). ^{1}H NMR (500 MHz, CDCl$_3$) δ 8.37 (d, *J* = 8.2 Hz, 1H), 7.88 (d, *J* = 7.9 Hz, 1H), 7.64 (t, *J* = 7.6 Hz, 1H), 7.53 (t, *J* = 7.3 Hz, 1H), 7.06 (d, *J* = 8.2 Hz, 1H), 6.96 (d, *J* = 7.8 Hz, 1H), 6.75 (s, 1H), 3.70 (m, 2H), 3.34 (m, 2H), 2.11 (m, 2H). ^{13}C NMR (126 MHz, CDCl$_3$) δ 168.96, 149.65, 143.72, 141.27, 129.19, 128.69, 127.08, 125.65, 122.01, 121.28, 120.44, 112.91, 106.64, 42.60, 31.76. ^{19}F NMR (376 MHz, CDCl$_3$) δ (-)136.63-(-)136.68 (m, 2F). ESI-MS (**m/z**): calcd for $C_{16}H_{13}BF_2N_2OSH^+$ 331.09, found $[M+H]^+$ 331.10.

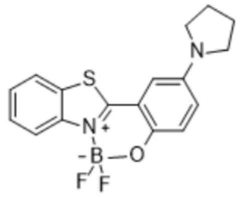

12B: red powder (69% yield). ^{1}H NMR (500 MHz, CDCl$_3$) δ 8.40 (d, *J* = 8.3 Hz, 1H), 7.90 (d, *J* = 8.0 Hz, 1H), 7.66 (t, *J* = 7.5 Hz, 1H), 7.58 – 7.52 (m, 1H), 7.15 (d, *J* = 8.9 Hz, 1H), 7.05 (s, 1H), 6.78 – 6.44 (m, 1H), 3.34 (m, 4H), 2.09 (m, 4H). ^{19}F NMR (376 MHz, CDCl$_3$) δ (-)136.90-(-)136.97 (m, 2F). ESI-MS (**m/z**): calcd for $C_{17}H_{15}BF_2N_2OSH^+$ 345.10, found $[M+H]^+$ 345.10.

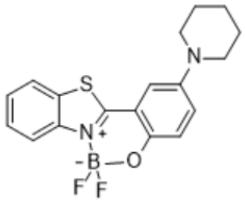

13B: yellow powder (64% yield). ^{1}H NMR (500 MHz, CDCl$_3$) δ 8.38 (d, *J* = 8.3 Hz, 1H), 7.90 (d, *J* = 8.0 Hz, 1H), 7.68 – 7.62 (m, 1H), 7.54 (dd, *J* = 11.3, 4.0 Hz, 1H), 7.34 (dd, *J* = 9.1, 2.8 Hz, 1H), 7.13 (d, *J* = 9.1 Hz, 1H), 7.07 (s, 1H), 3.14 – 3.06 (m, 4H), 1.82 – 1.74 (m, 4H), 1.61 (m, 2H). ^{13}C NMR (126 MHz, CDCl$_3$) δ 169.11, 150.97, 146.03, 143.70, 129.36, 129.14, 128.69, 127.12, 126.73, 122.00, 120.94, 120.45, 112.70, 52.10, 25.82, 23.84. ^{19}F NMR (376 MHz, CDCl$_3$) δ (-)135.95-(-)136.03 (m, 2F). ESI-MS (**m/z**): calcd for $C_{18}H_{17}BF_2N_2OSH^+$ 359.12, found $[M+H]^+$ 359.14.

2 Supplementary Tables

Table S1. Optical properties of compounds 1-10 and 1B-10B in EtOAc

compds	λ_{abs} (nm)	ε ($M^{-1}cm^{-1}$)	λ_{em} (nm)	λ_{ex} (nm)	stokes shift (nm)	Φ_F
**1**[a]	368	-	562	370	192	0.15
**1B**[a]	-	-	-	-	-	-
**2**	328	17 600	511	338	173	0.09
**2B**	343	8 770	422	360	62	0.15
**3**	335	22 000	414	363	51	0.05
**3B**	358	17 300	405	354	51	0.21
**4**	336	29 300	430	385	45	0.03
**4B**	361	12 800	407	360	47	0.79
**5**	356	22 500	394	342	52	0.07
**5B**	396	17 000	414	393	21	0.91
**6**	331	18 500	499	334	165	0.17
**6B**[a]	-	-	-	-	-	-
**7**	342	15 100	412	362	50	0.04
**7B**	358	14 200	500	317	183	0.18
**8**	343	14 900	370	360	10	0.04
**8B**	362	4 740	434	376	58	0.15
**9**	361	14 300	401	342	59	0.09
**9B**	381	6 560	479	392	87	0.31
**10**	350	4 620	431	361	70	0.03
**10B**	384	878	590	441	149	0.10

[a] Not detected due to poor solubility.

3 Supplementary figures

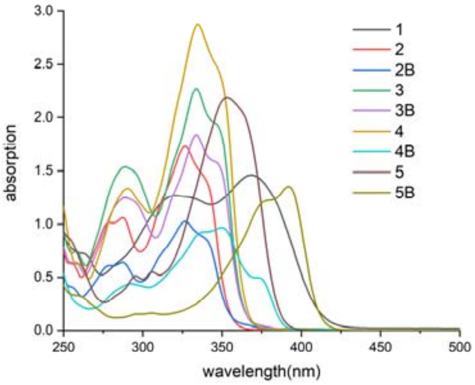

Supplementary Figure 1. Absorption spectra of compounds 1-5, 2B-5B in CH_3CN.

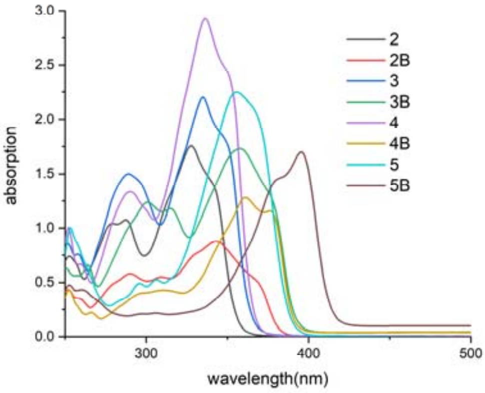

Supplementary Figure 2. Absorption spectra of compounds 2-5, 2B-5B in EtOAc.

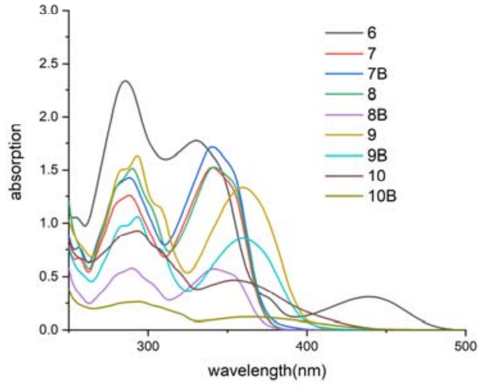

Supplementary Figure 3. Absorption spectra of compounds 6-10, 7B-10B in CH_3CN.

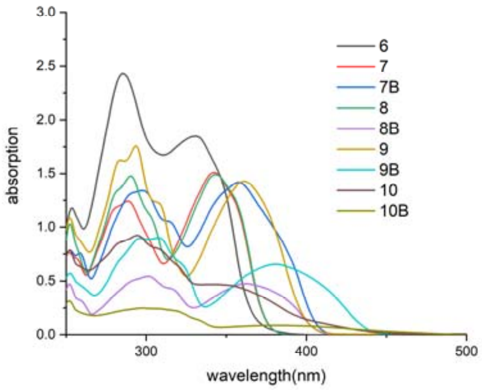

Supplementary Figure 4. Absorption spectra of compounds 6-10, 7B-10B in EtOAc.

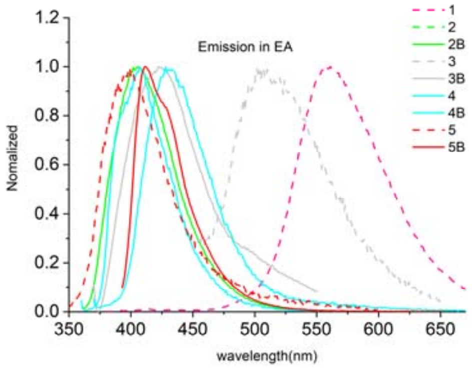

Supplementary Figure 5. Emission spectra of compounds 1-5, 2B-5B in EtOAc.

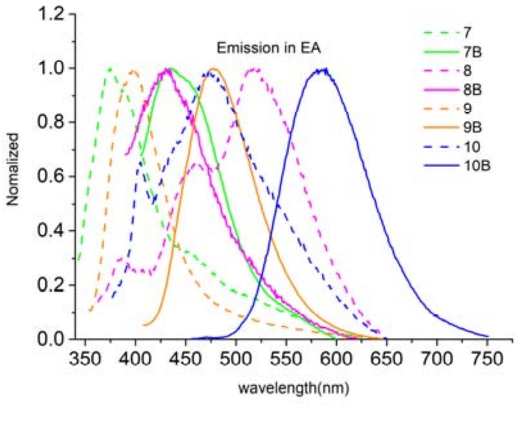

Supplementary Figure 6. Emission spectra of compounds 7-10, 7B-10B in EtOAc.

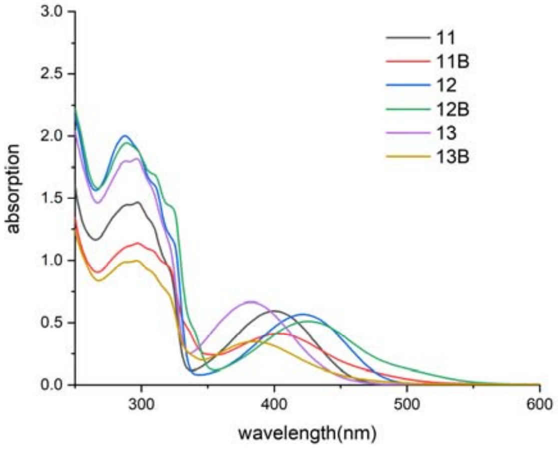

Supplementary Figure 7. Absorption spectra of compounds 11-13, 11B-13B in CH_3CN.

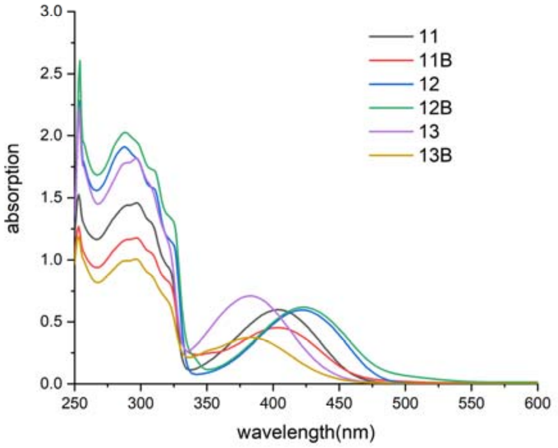

Supplementary Figure 8. Absorption spectra of compounds 11-13, 11B-13B in EtOAc.

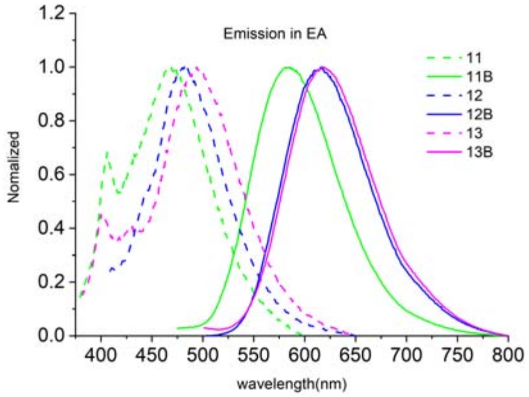

Supplementary Figure 9. Emission spectra of compounds 11-13, 11B-13B in EtOAc.

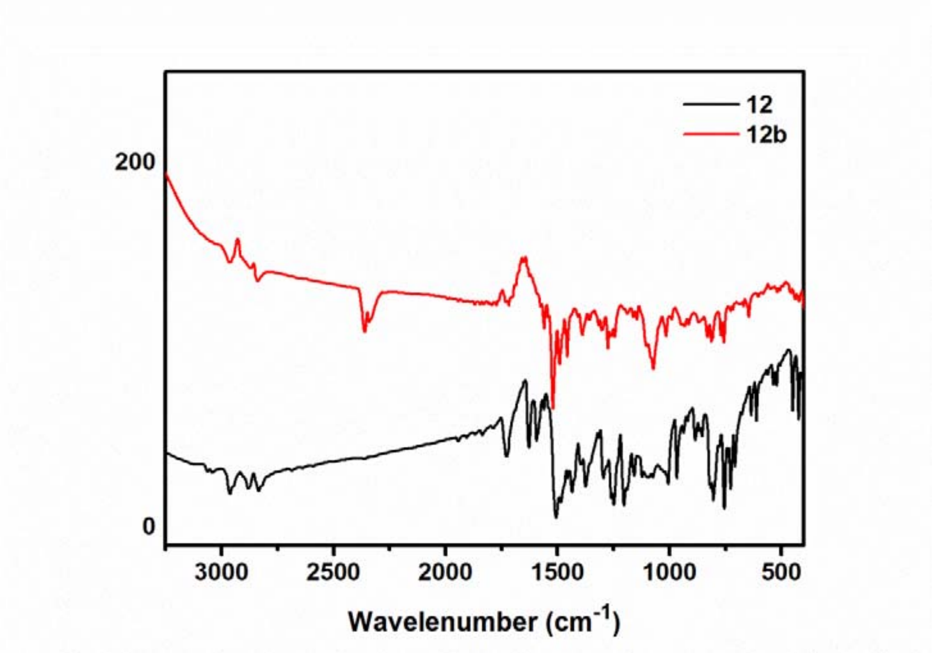

Supplementary Figure 10. IR emission spectra of 12B in comparison to that of 12.

4 NMR spectra traces

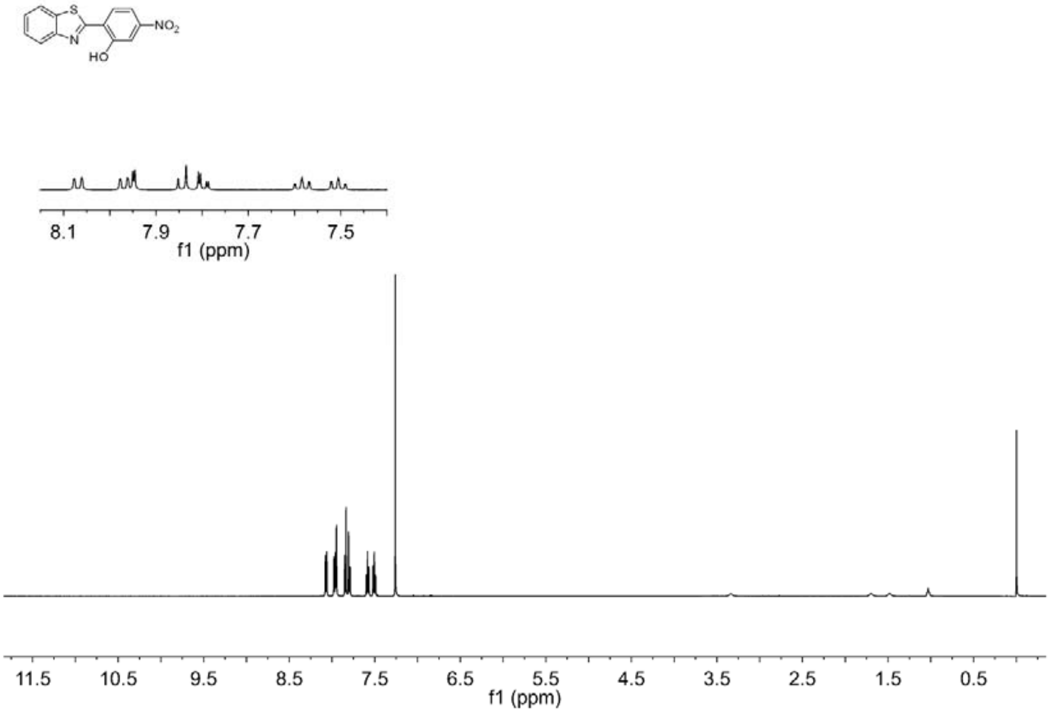

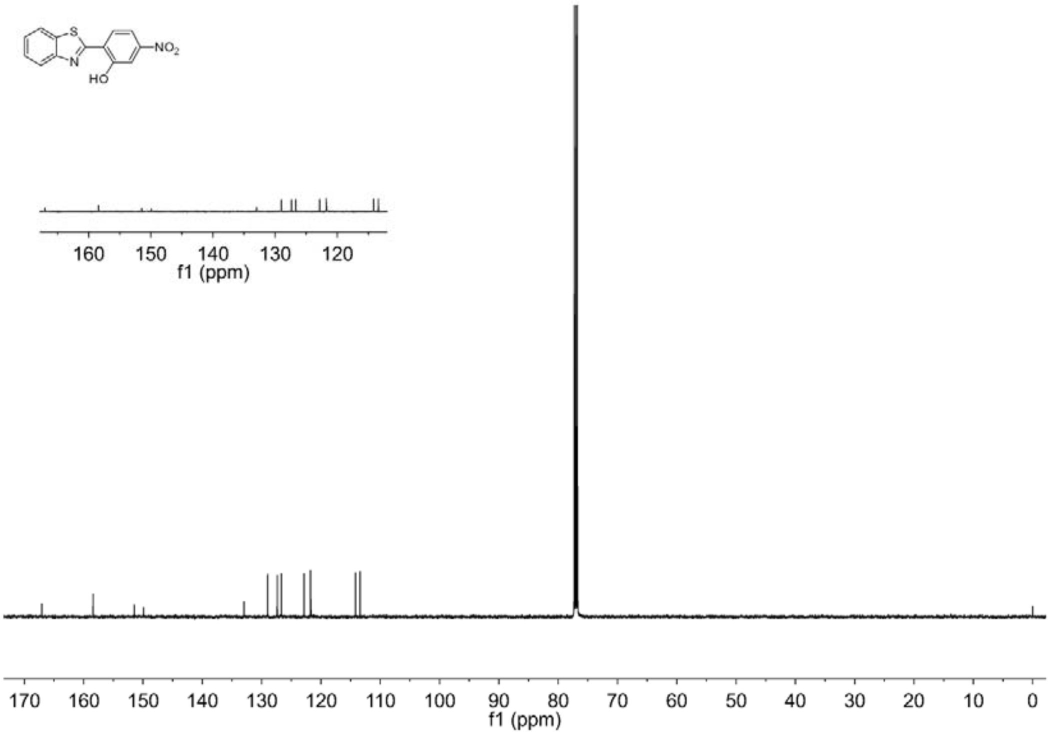

^{1}H NMR, ^{13}C NMR spectra of **1** in chloroform-*d*.

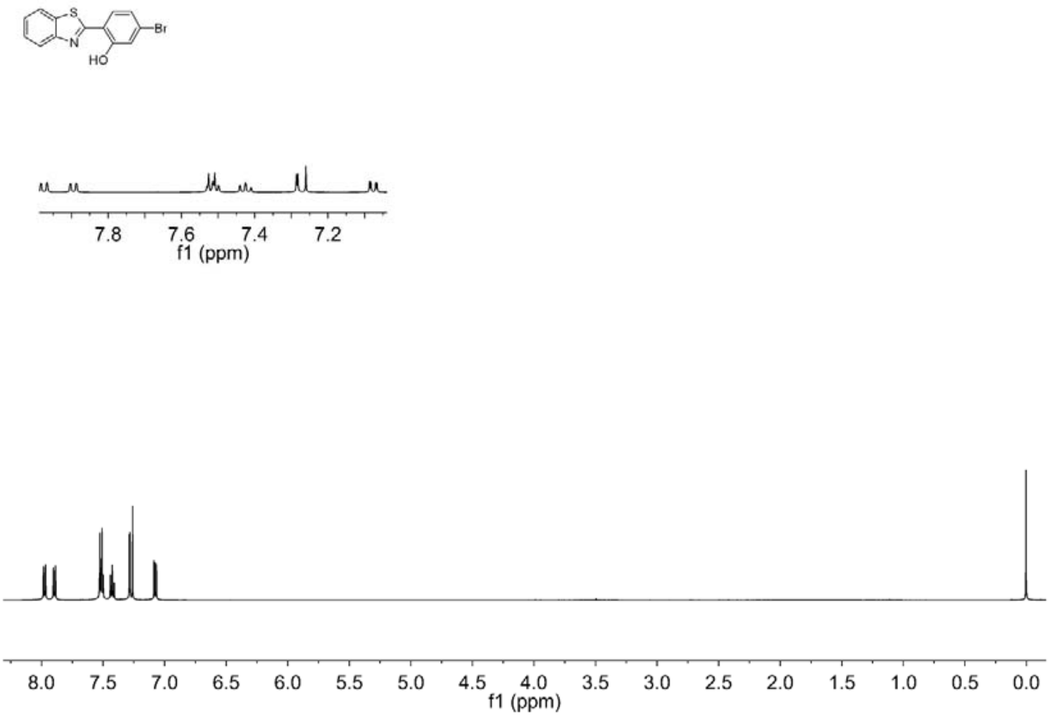

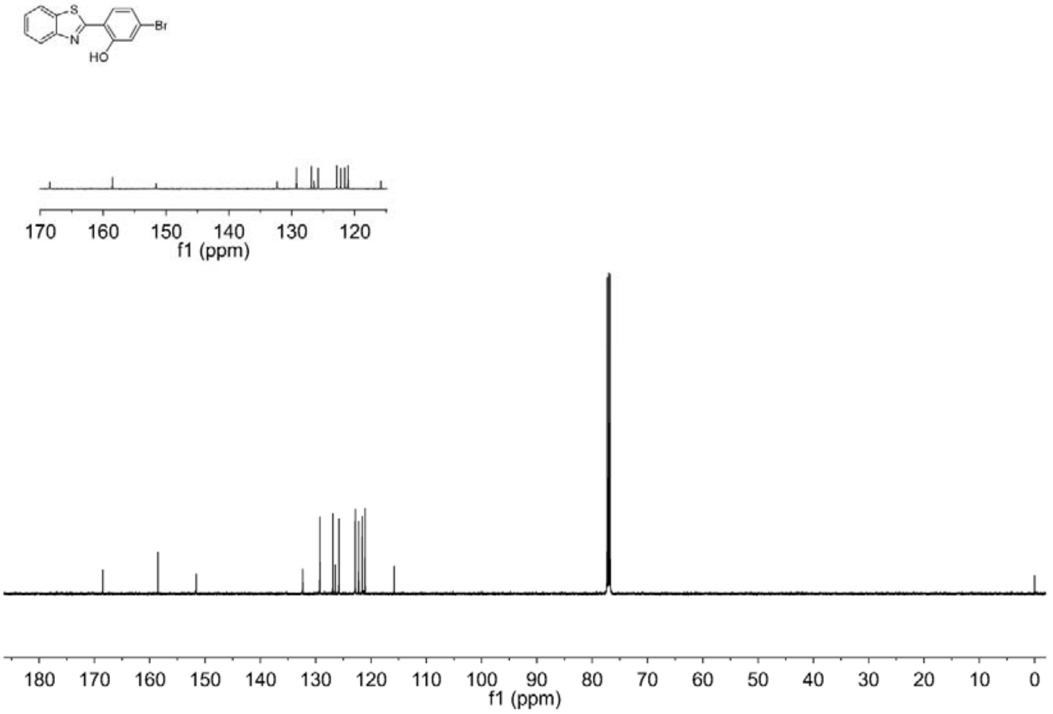

^{1}H NMR, ^{13}C NMR spectra of **2** in chloroform-*d*.

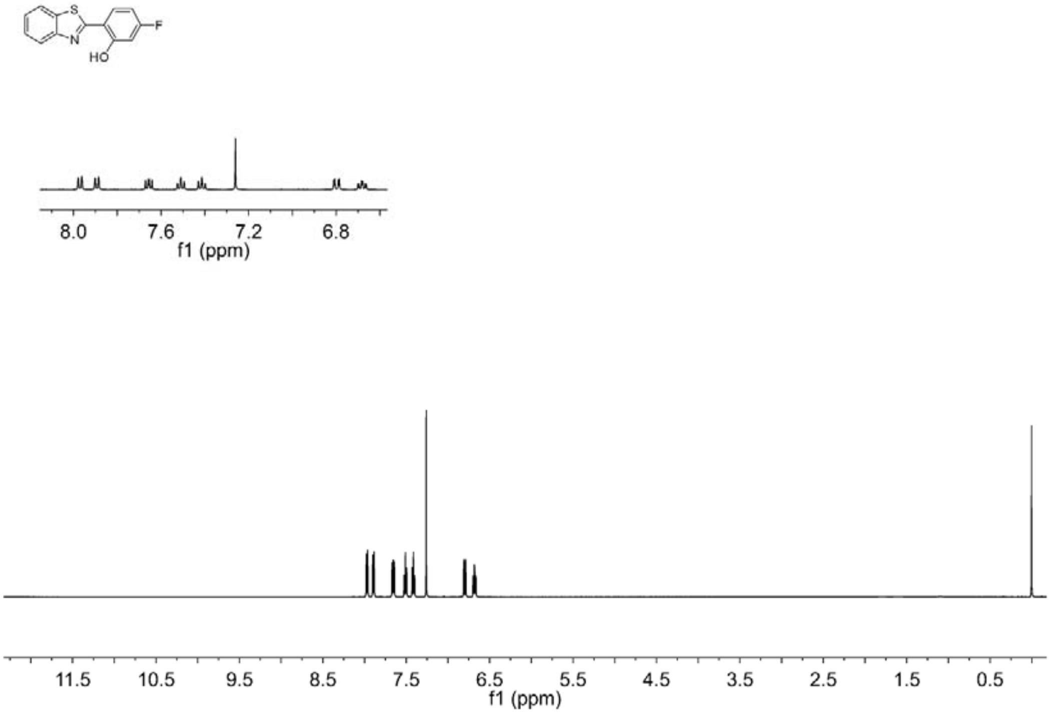

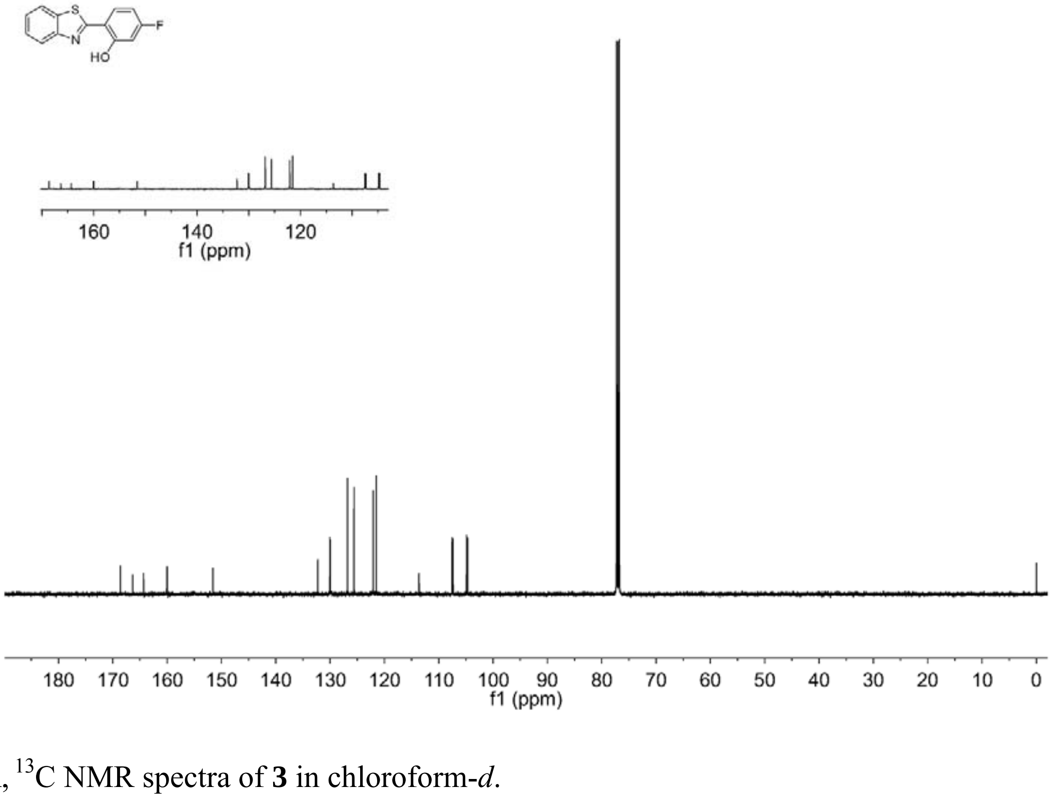

^{1}H NMR, ^{13}C NMR spectra of **3** in chloroform-*d*.

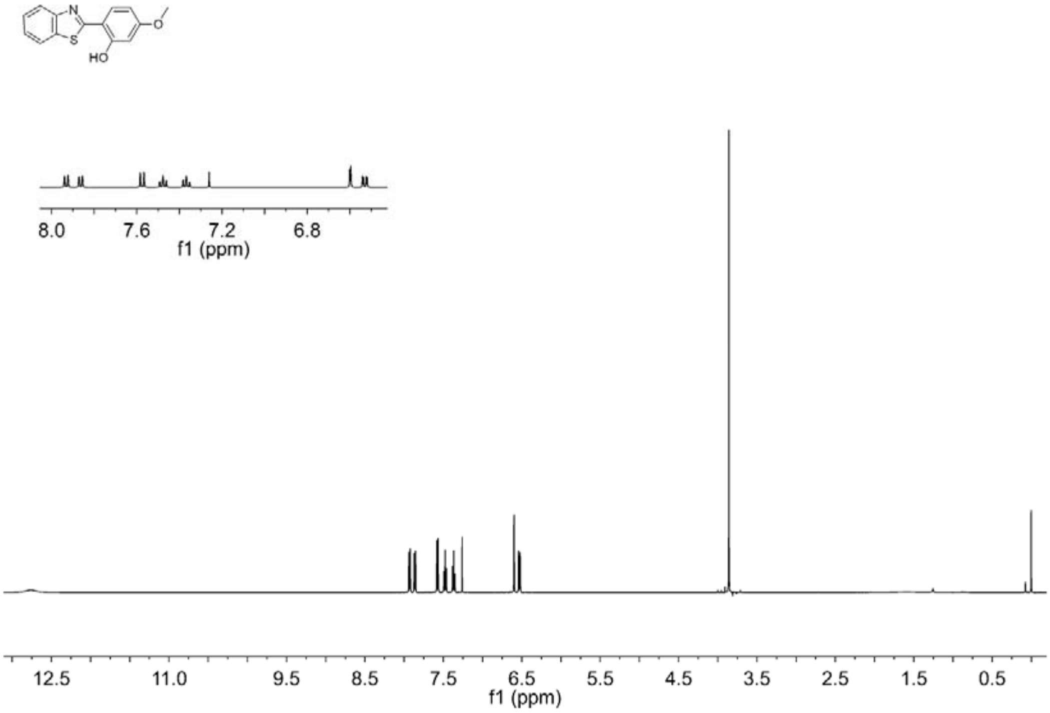

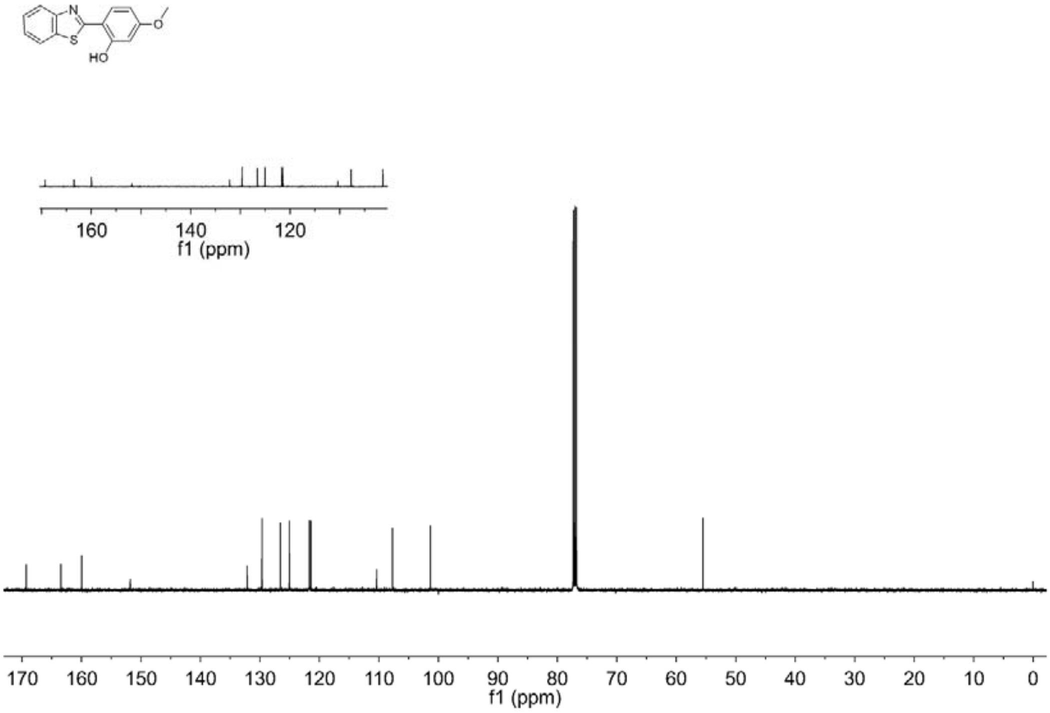

^{1}H NMR, ^{13}C NMR spectra of **4** in chloroform-*d*.

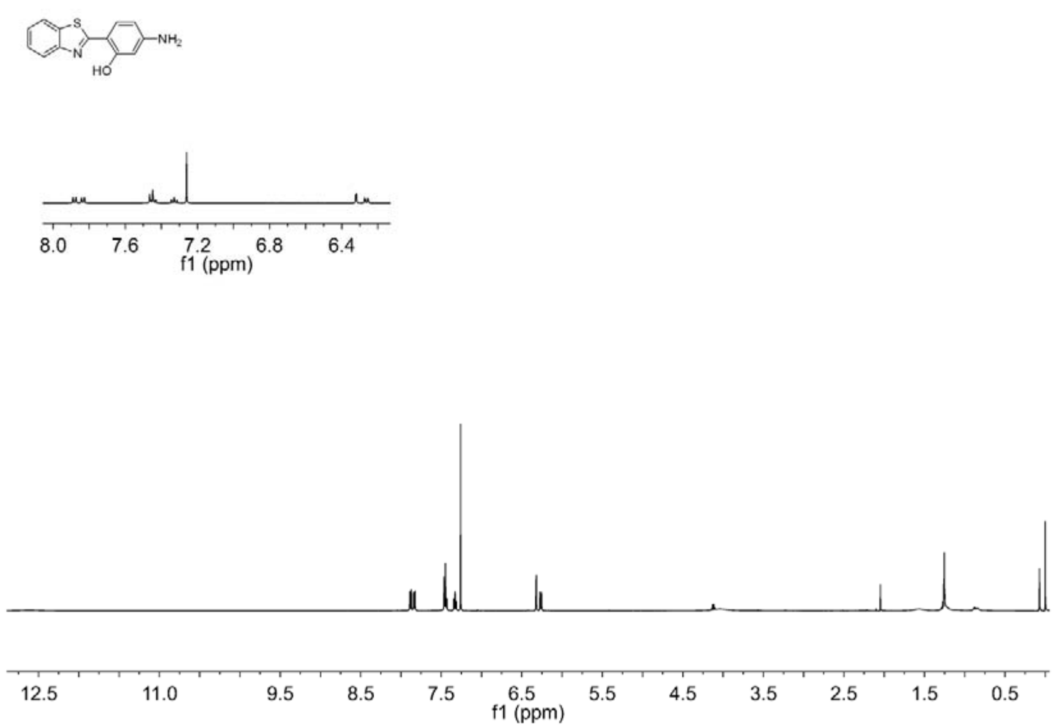

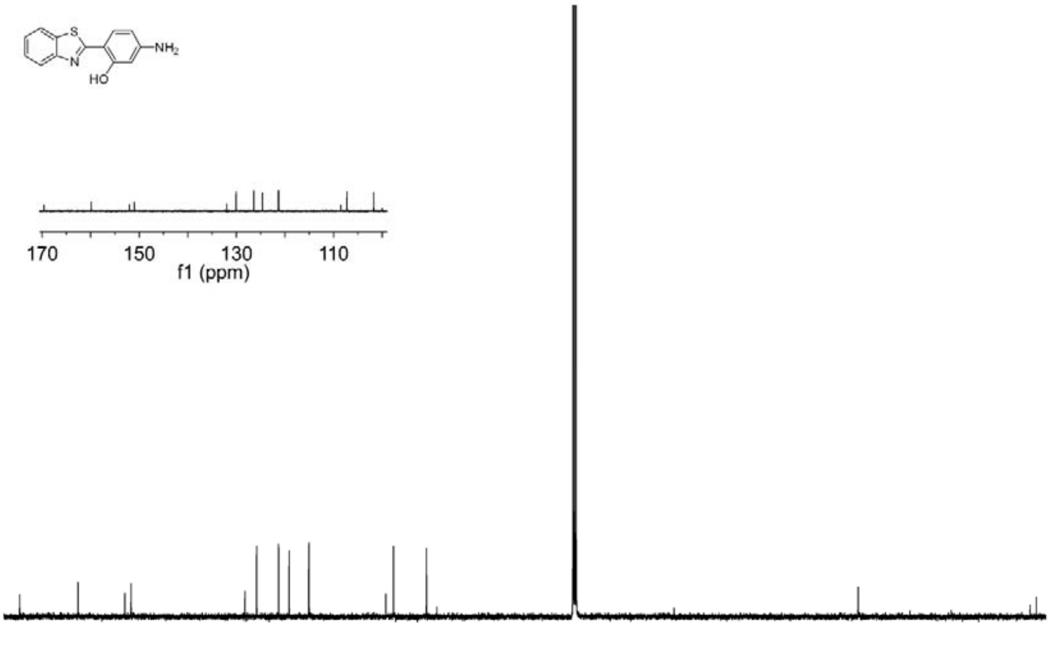

^{1}H NMR, ^{13}C NMR spectra of **5** in chloroform-*d*.

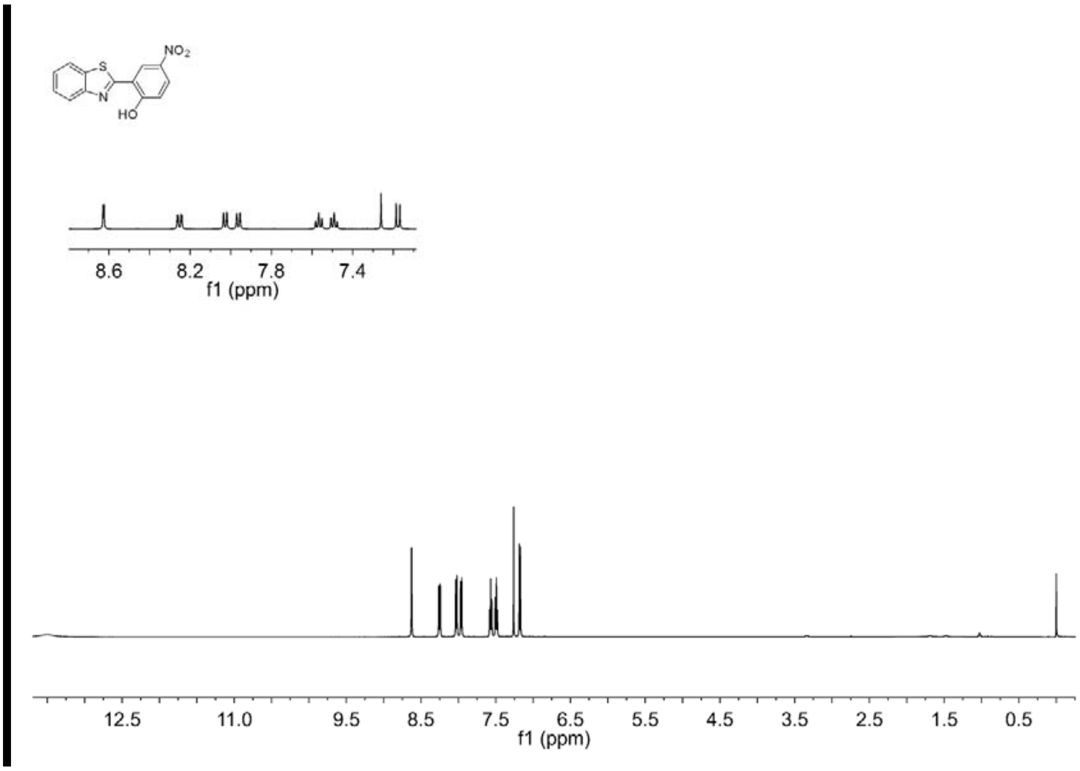

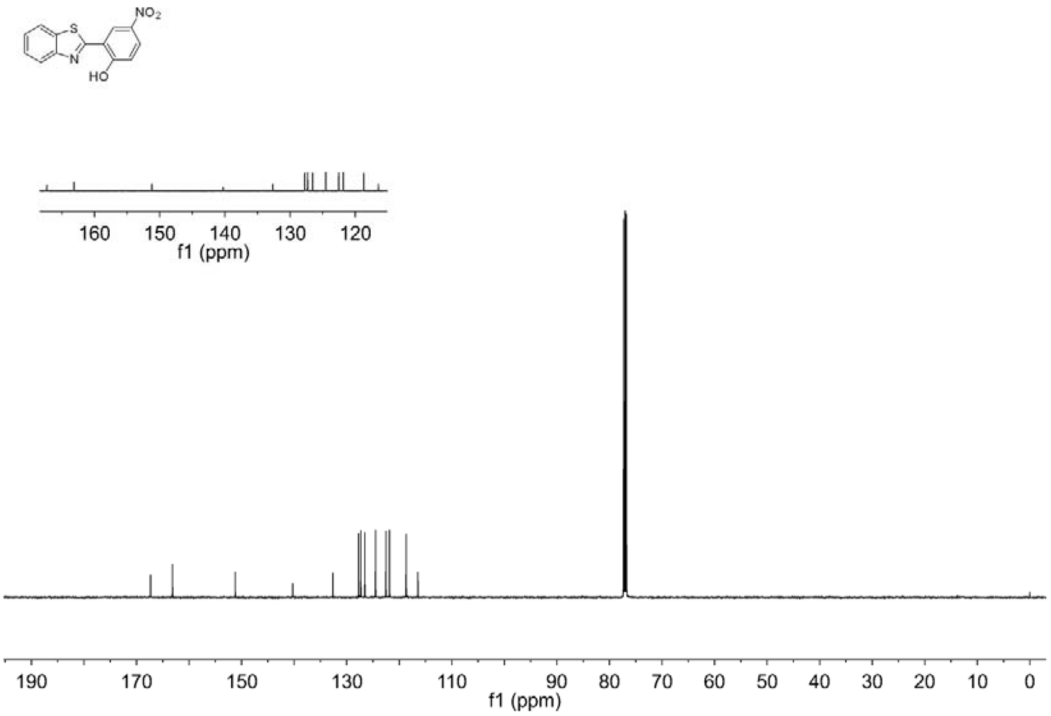

^{1}H NMR, ^{13}C NMR spectra of **6** in chloroform-*d*.

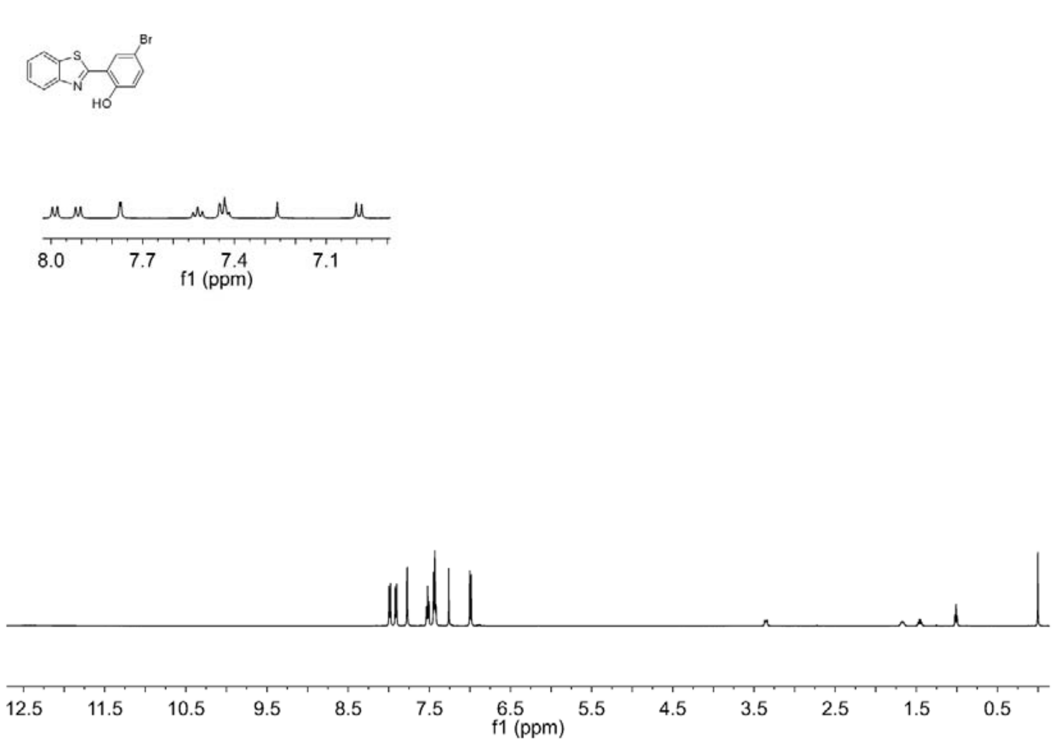

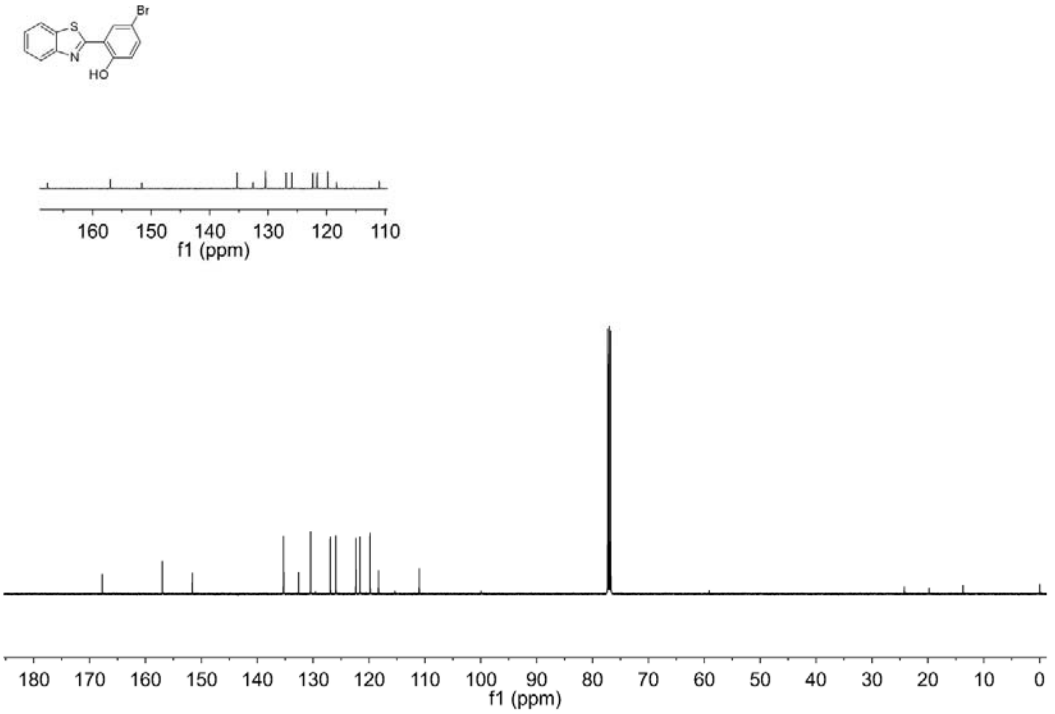

^{1}H NMR, ^{13}C NMR spectra of **7** in chloroform-*d*.

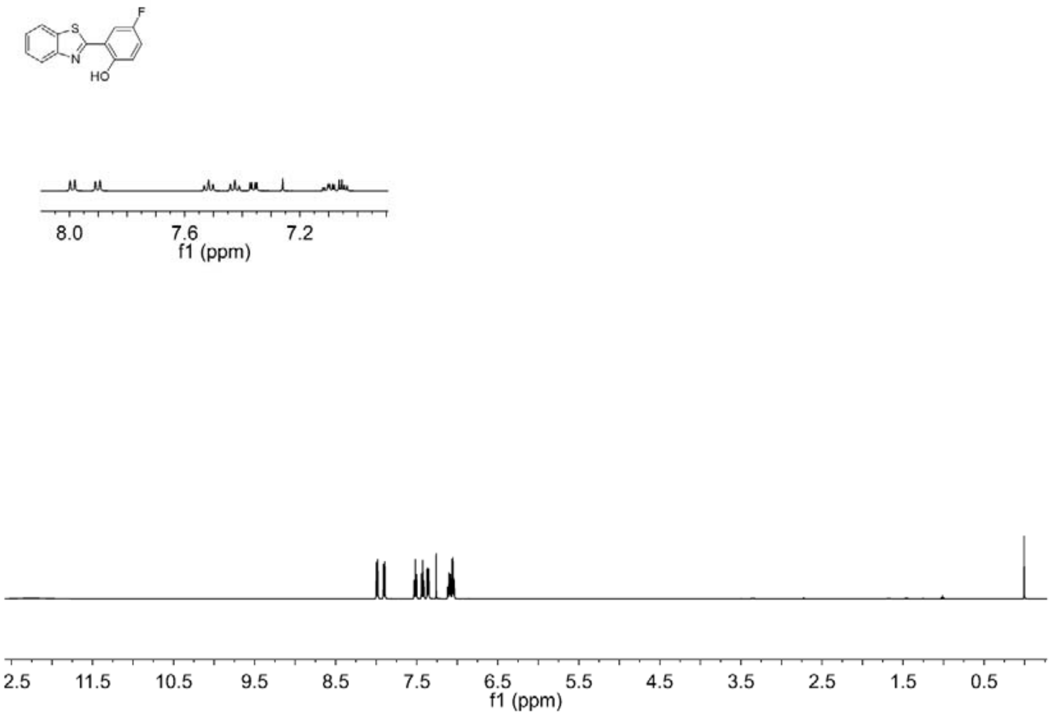

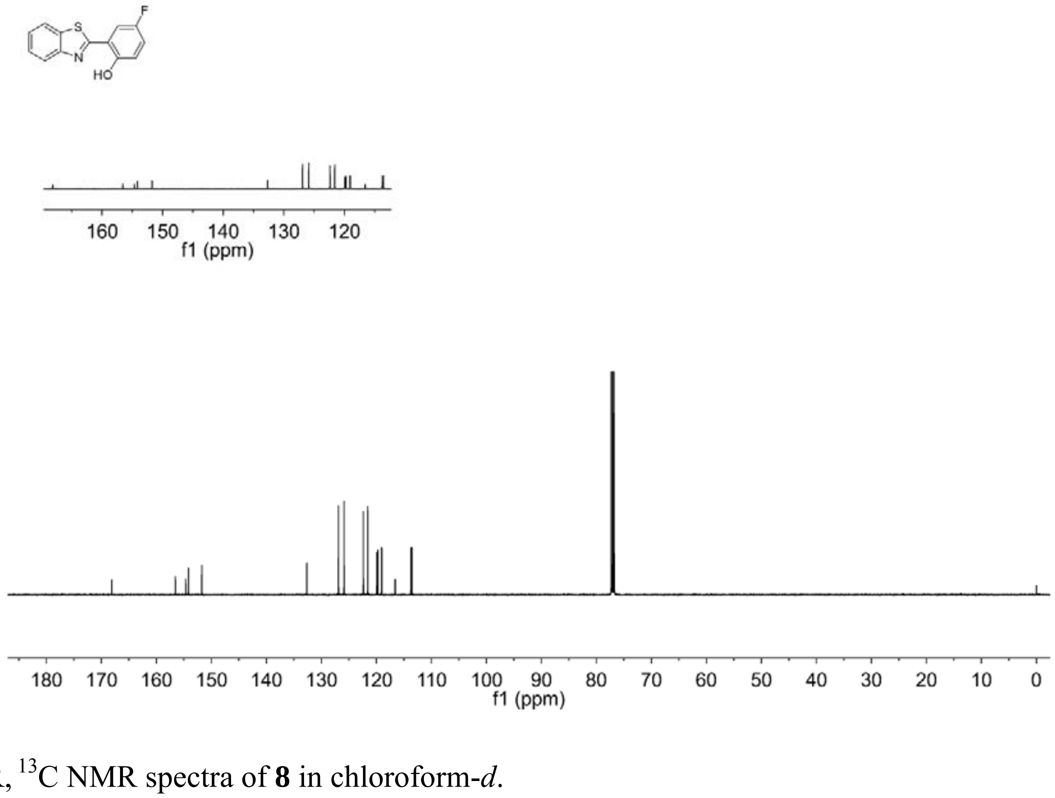

^{1}H NMR, ^{13}C NMR spectra of **8** in chloroform-*d*.

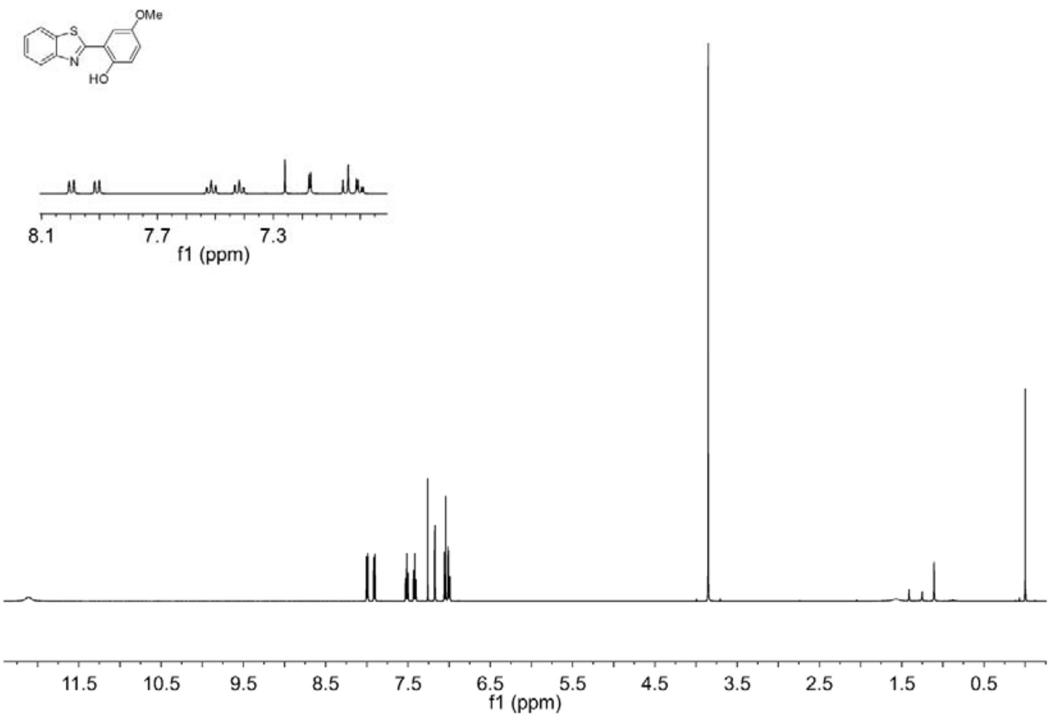

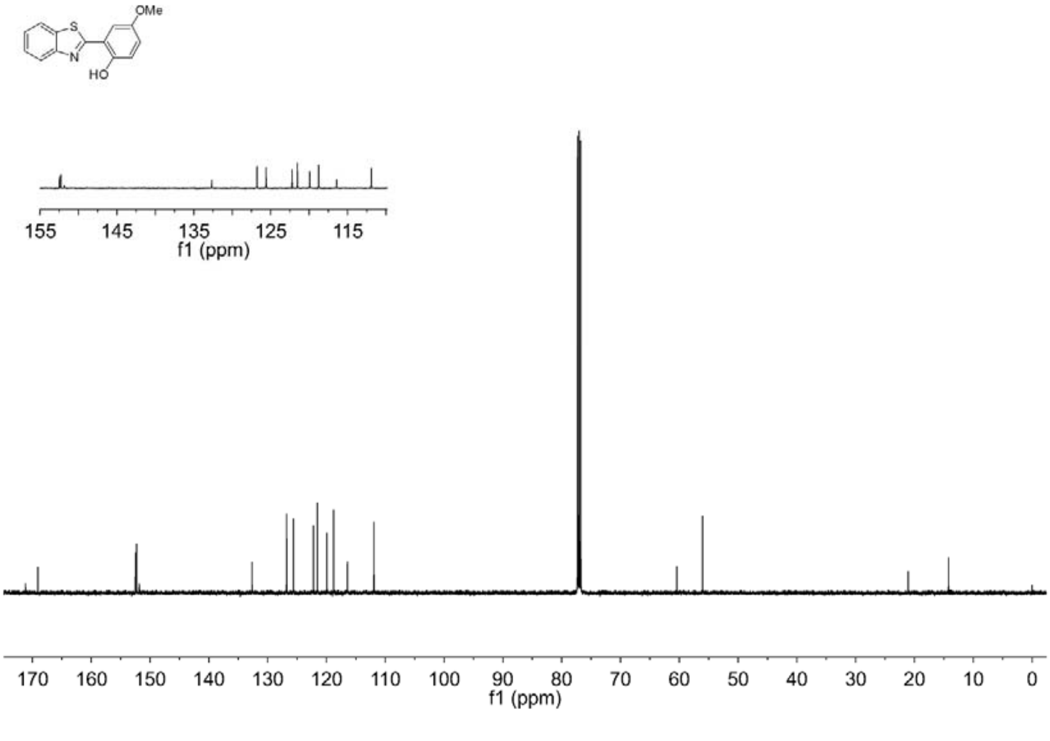

^{1}H NMR, ^{13}C NMR spectra of **9** in chloroform-*d*.

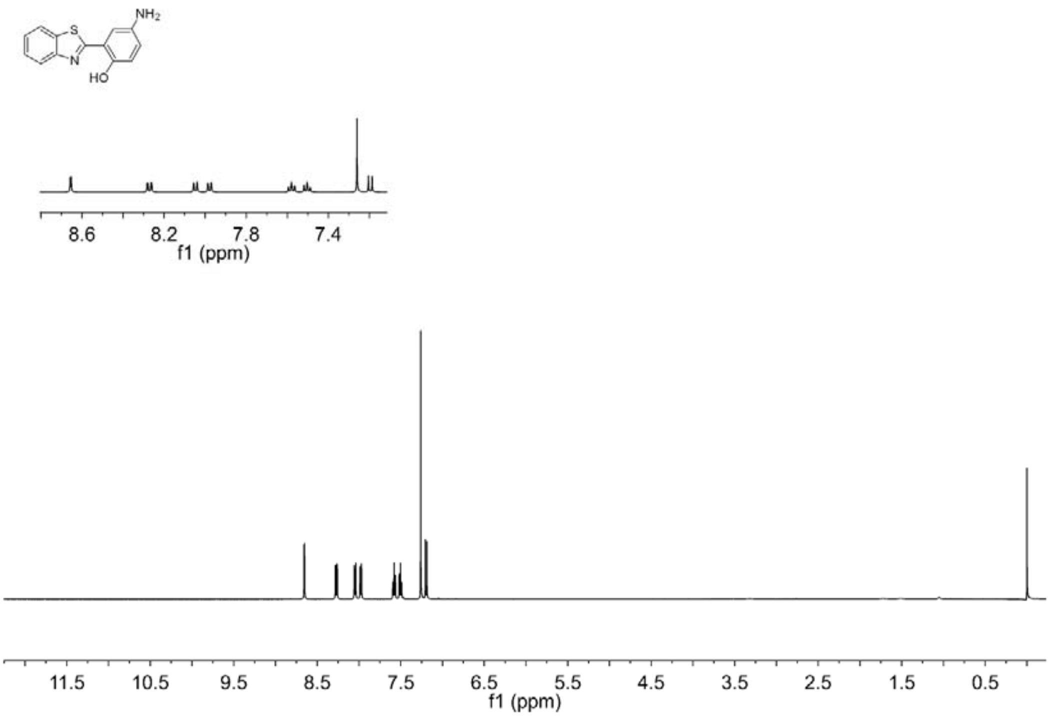

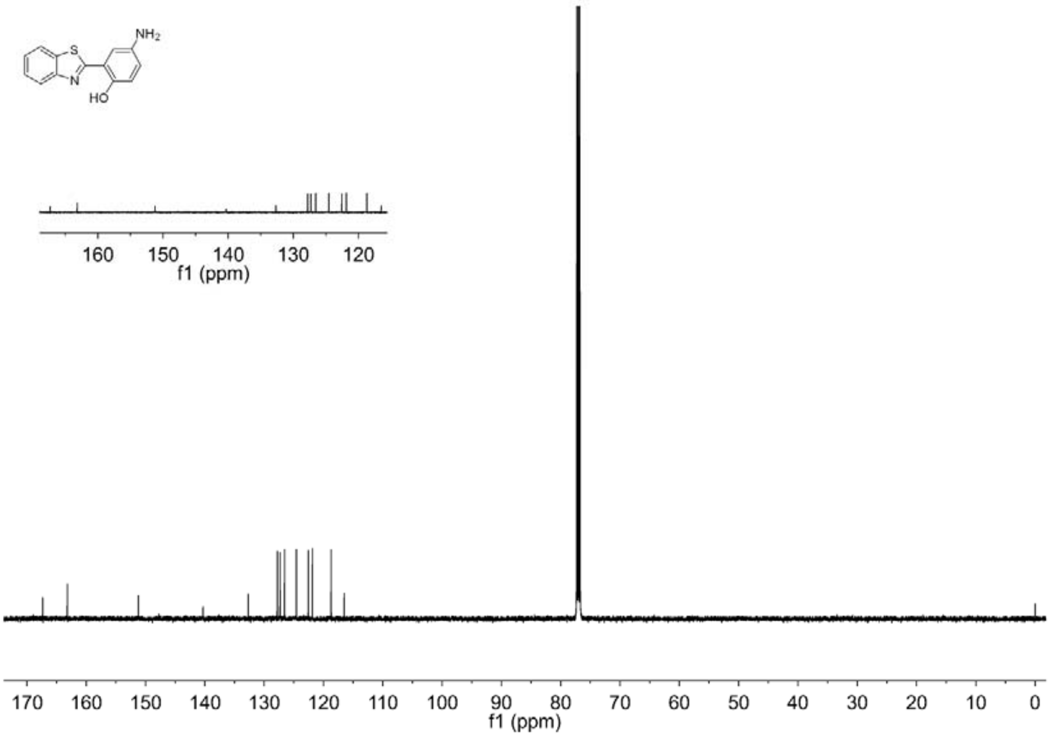

^{1}H NMR, ^{13}C NMR spectra of **10** in chloroform-*d*.

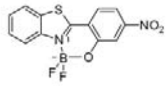

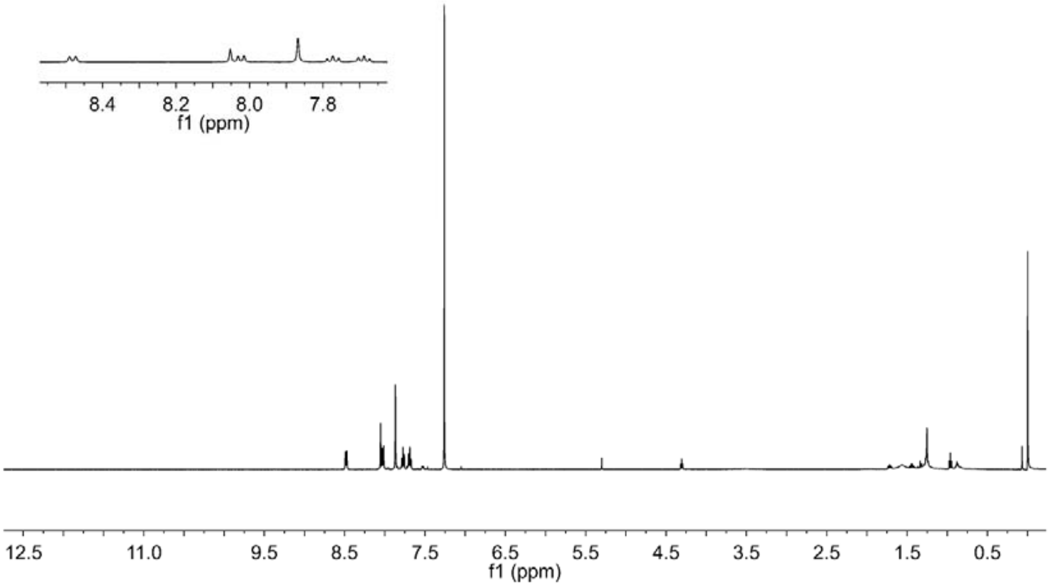

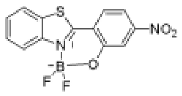

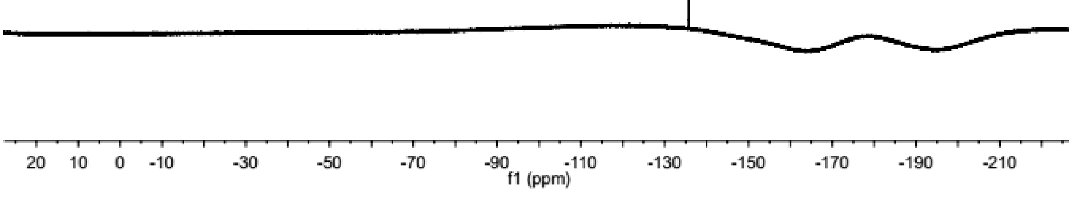

^{1}H NMR, ^{19}F NMR spectra of **1B** in chloroform-*d*.

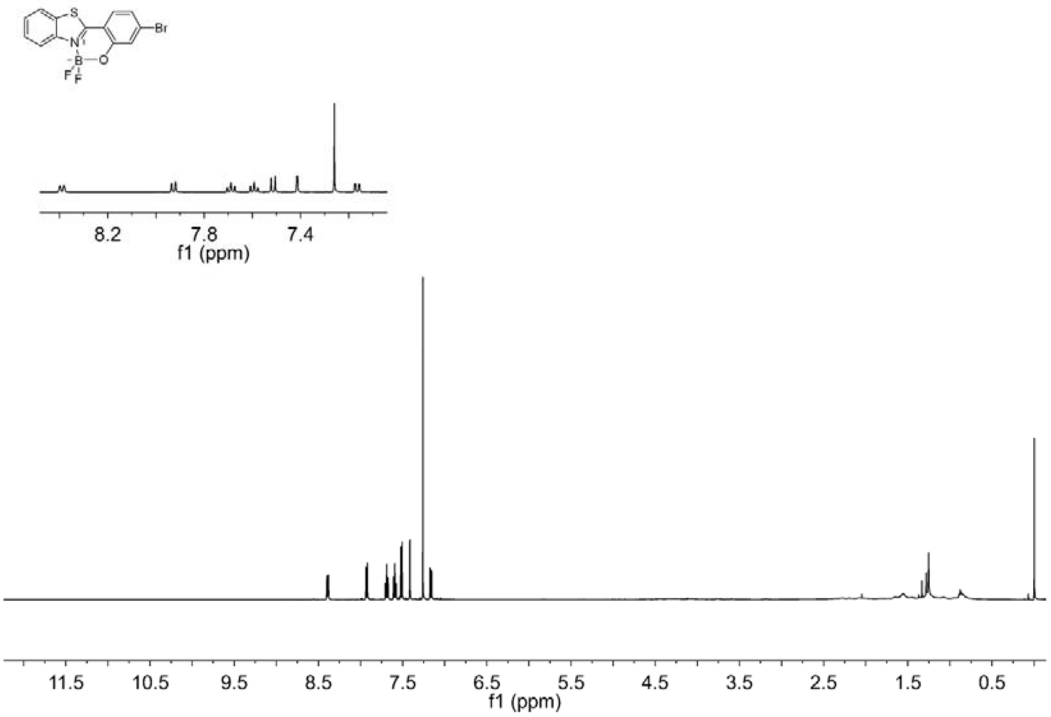
Br
8.2
7.8
7.4
f1 (ppm)
11.5
10.5
9.5
8.5
7.5
6.5
5.5
4.5
3.5
2.5
1.5
0.5
f1 (ppm)

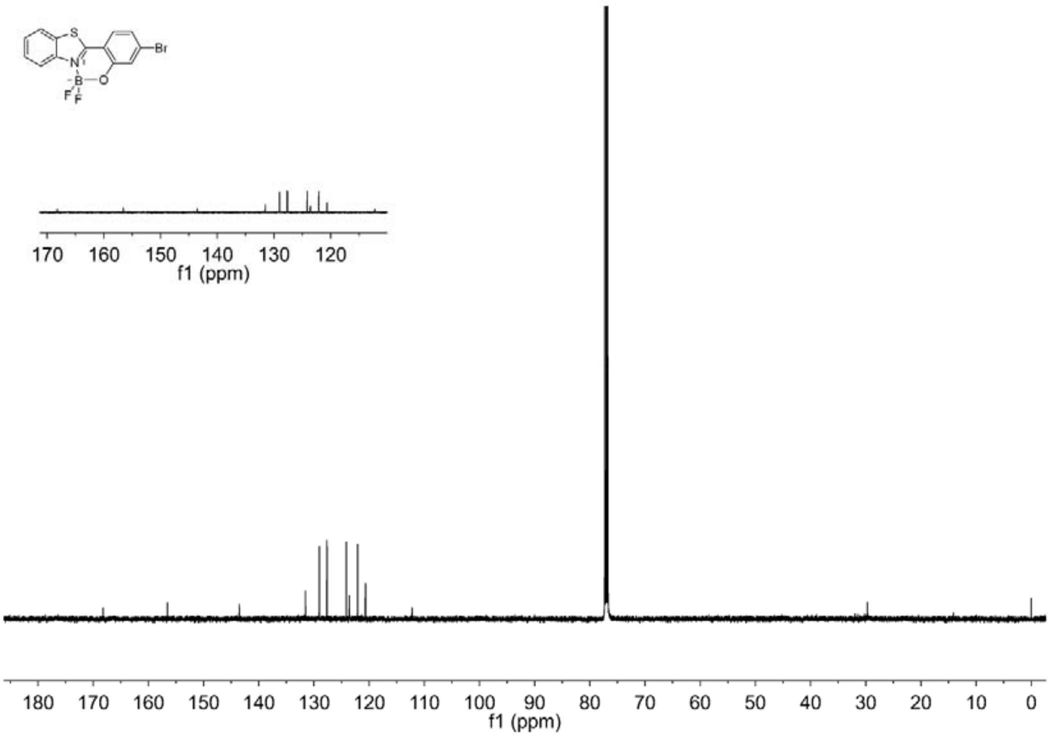
Br
170
160
150
140
130
120
f1 (ppm)
180
170
160
150
140
130
120
110
100
90
80
70
60
50
40
30
20
10
0
f1 (ppm)

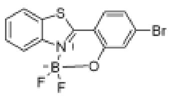

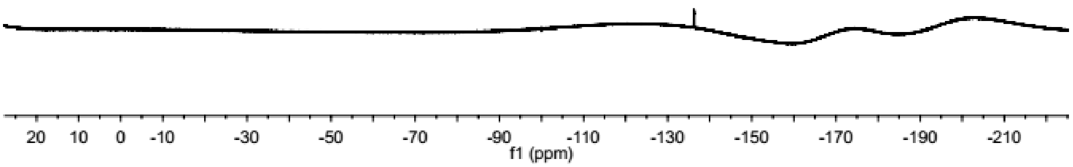

^{1}H NMR, ^{13}C NMR, ^{19}F NMR spectra of **2B** in chloroform-*d*.

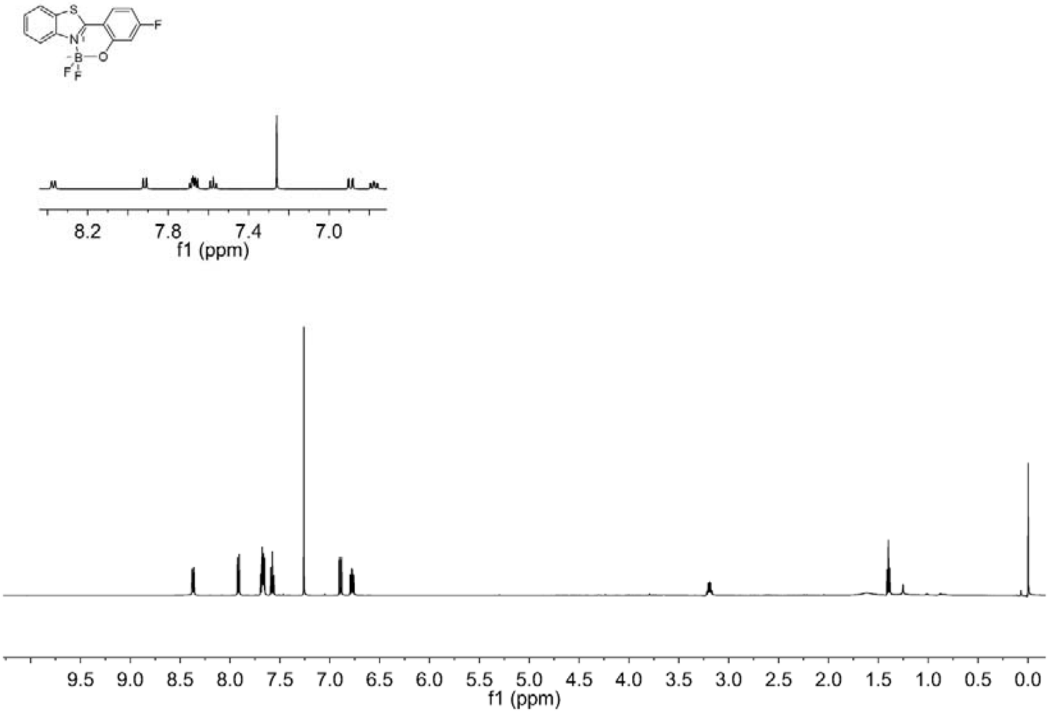
S
N
B
O
F
F
F
8.2
7.8
7.4
7.0
f1 (ppm)
9.5
9.0
8.5
8.0
7.5
7.0
6.5
6.0
5.5
5.0
4.5
4.0
3.5
3.0
2.5
2.0
1.5
1.0
0.5
0.0
f1 (ppm)

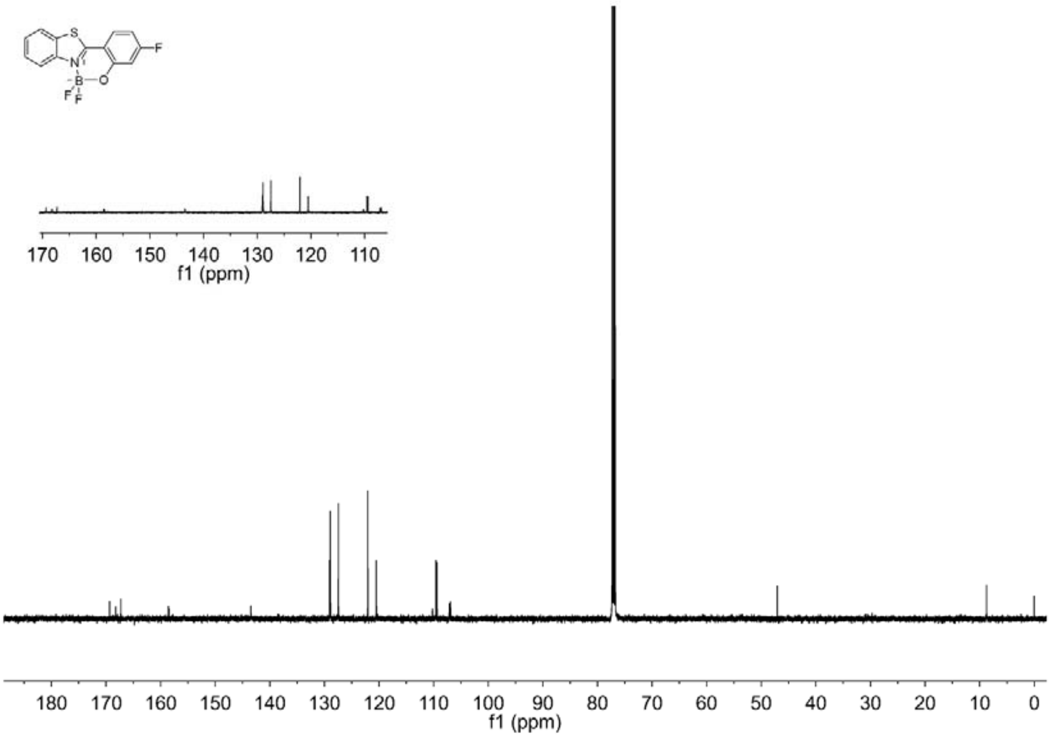
S
N
B
O
F
F
F
170
160
150
140
130
120
110
f1 (ppm)
180
170
160
150
140
130
120
110
100
90
80
70
60
50
40
30
20
10
0
f1 (ppm)

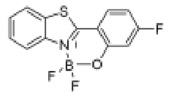

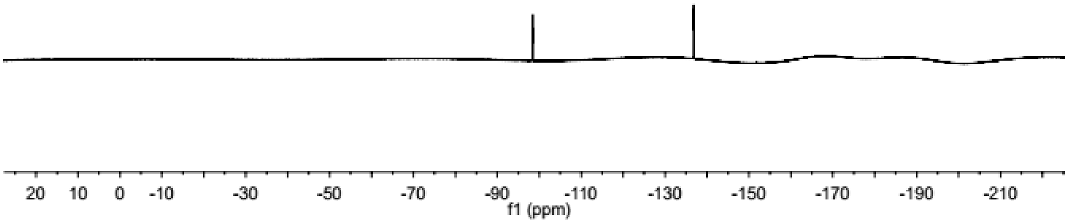

^{1}H NMR, ^{13}C NMR, ^{19}F NMR spectra of **3B** in chloroform-*d*.

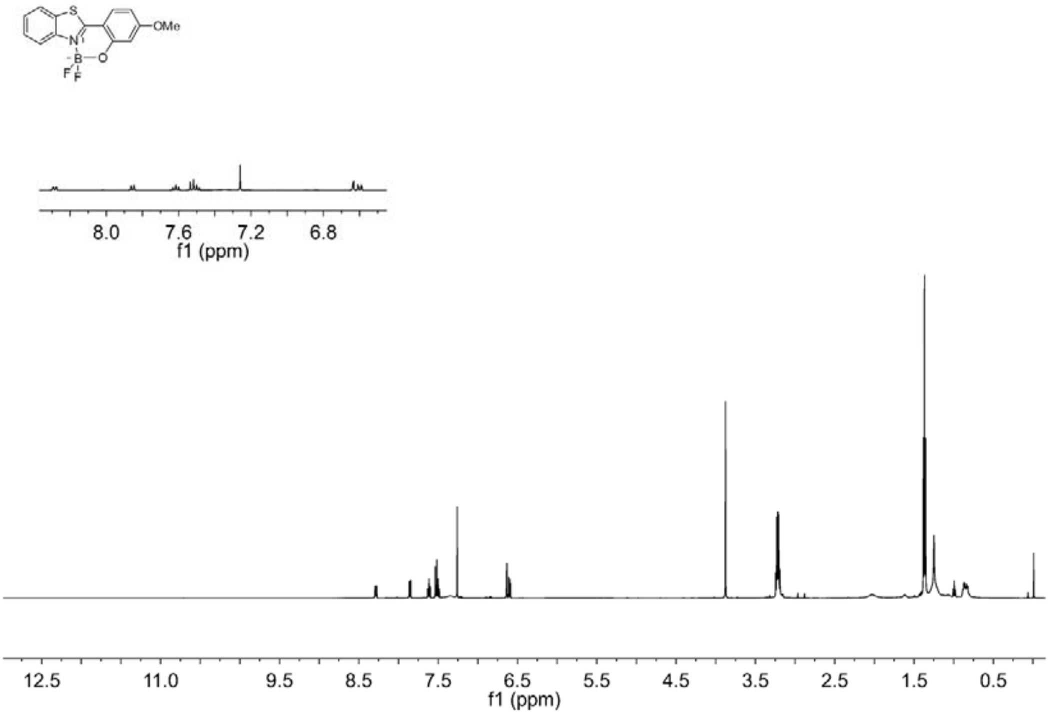
S
N
B
O
F F
OMe
8.0 7.6 7.2 6.8
f1 (ppm)
12.5 11.0 9.5 8.5 7.5 6.5 5.5 4.5 3.5 2.5 1.5 0.5
f1 (ppm)

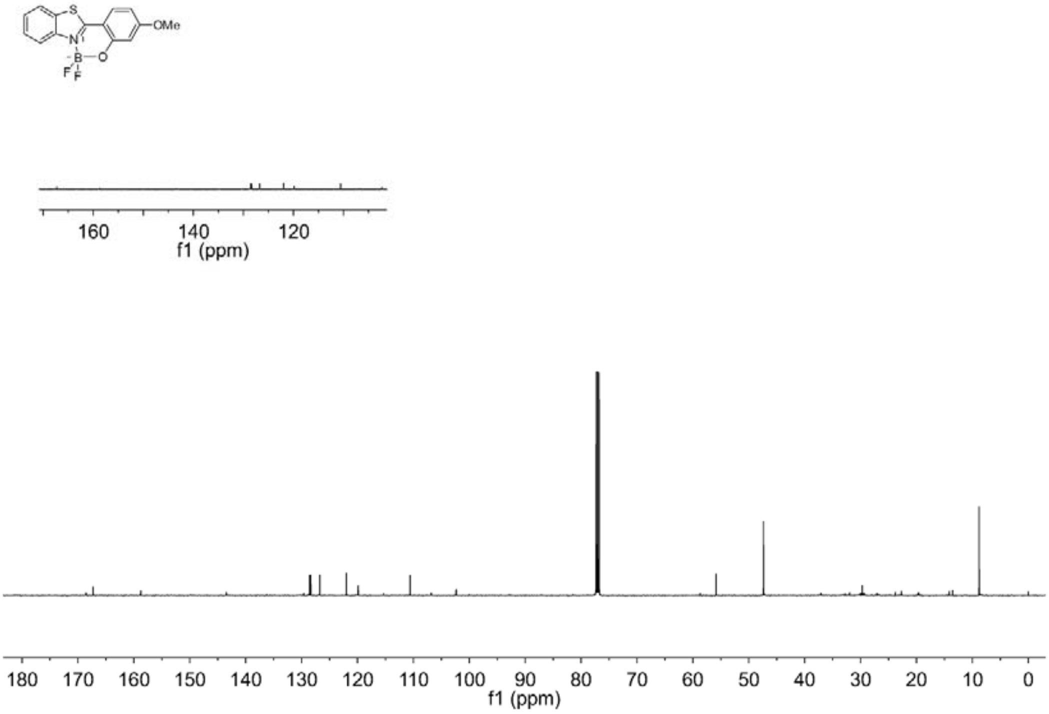
S
N
B
O
F F
OMe
160 140 120
f1 (ppm)
180 170 160 150 140 130 120 110 100 90 80 70 60 50 40 30 20 10 0
f1 (ppm)

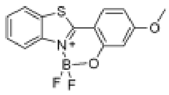

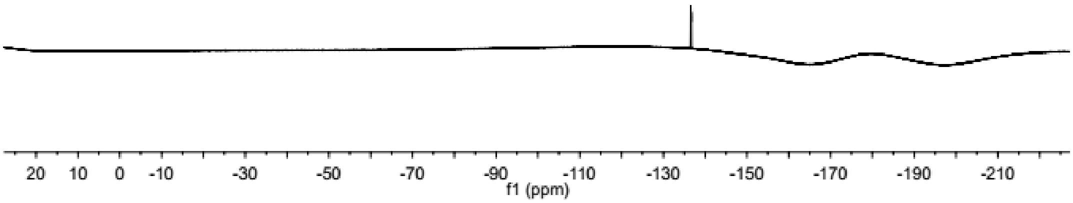

^{1}H NMR, ^{13}C NMR, ^{19}F NMR spectra of **4B** in chloroform-*d*.

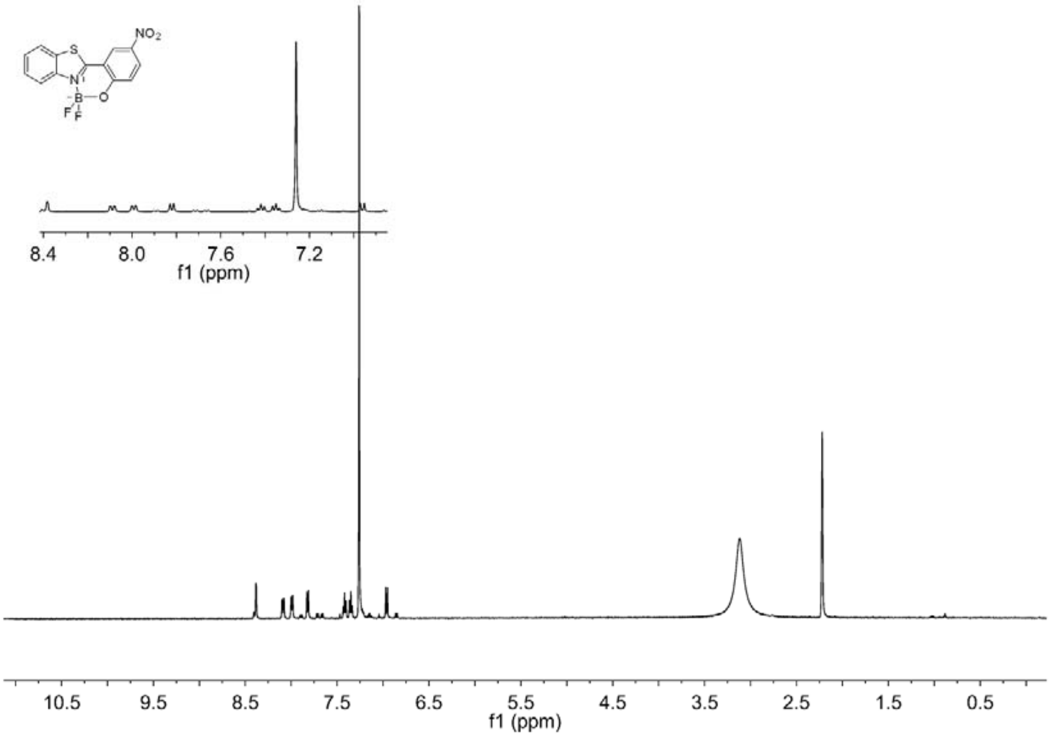

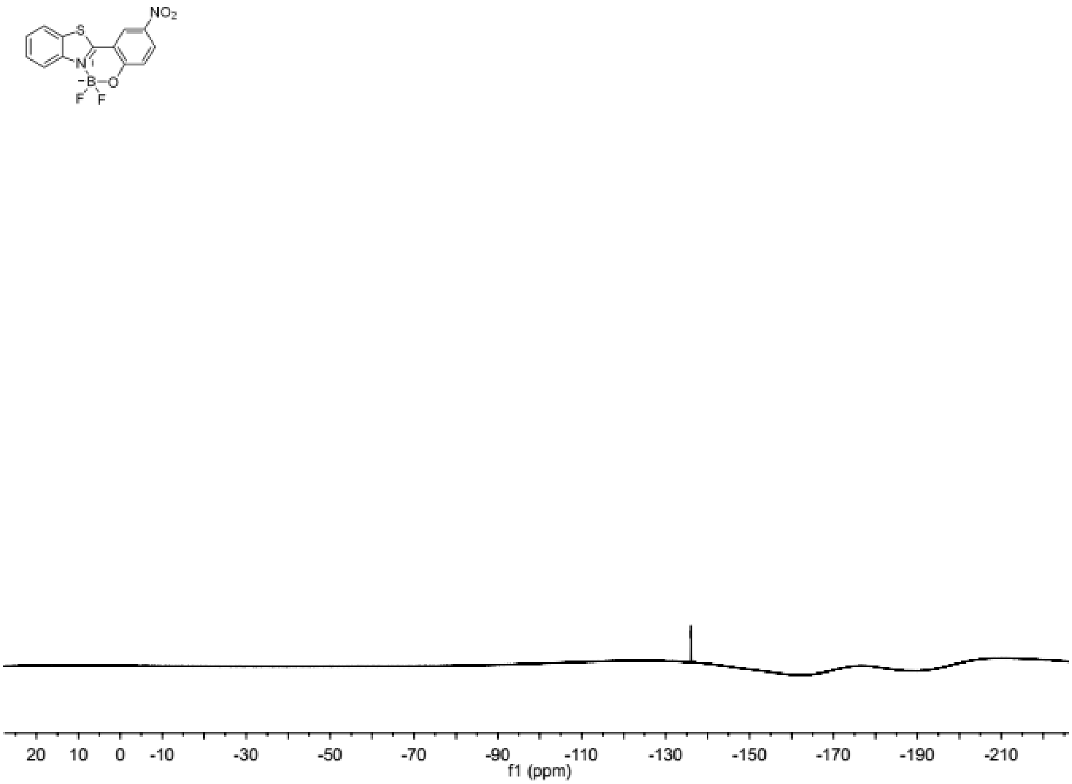

^{1}H NMR, ^{19}F NMR spectra of **6B** in chloroform-*d*.

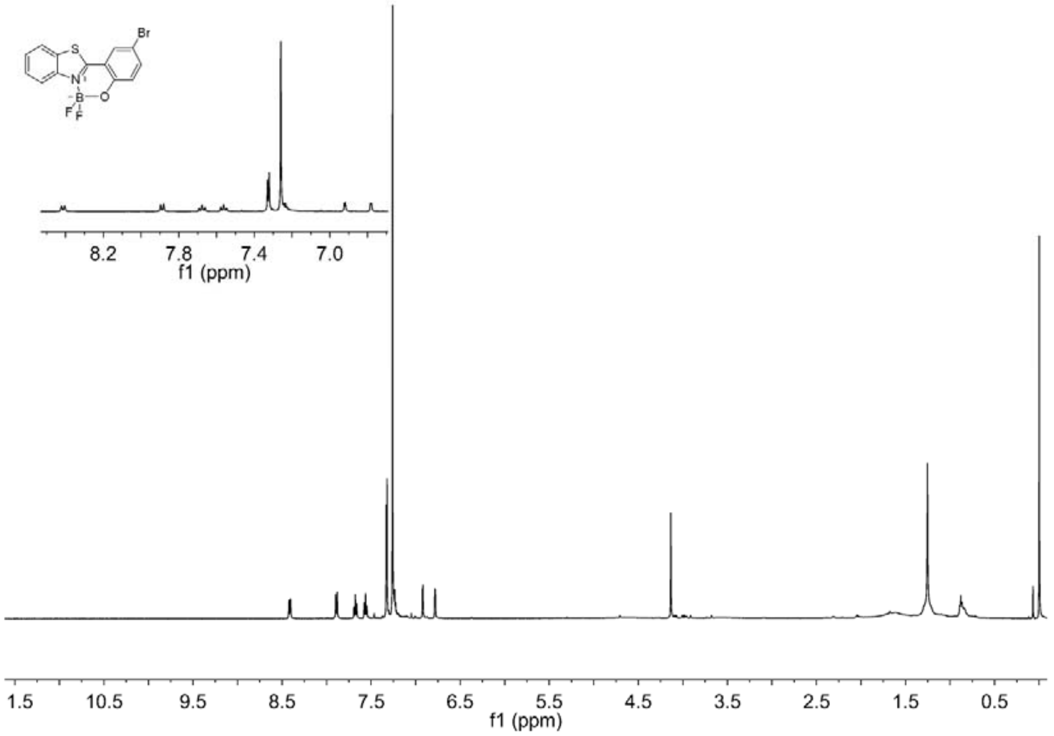
Br
S
N
B
O
F
F
8.2 7.8 7.4 7.0
f1 (ppm)
1.5 10.5 9.5 8.5 7.5 6.5 5.5 4.5 3.5 2.5 1.5 0.5
f1 (ppm)

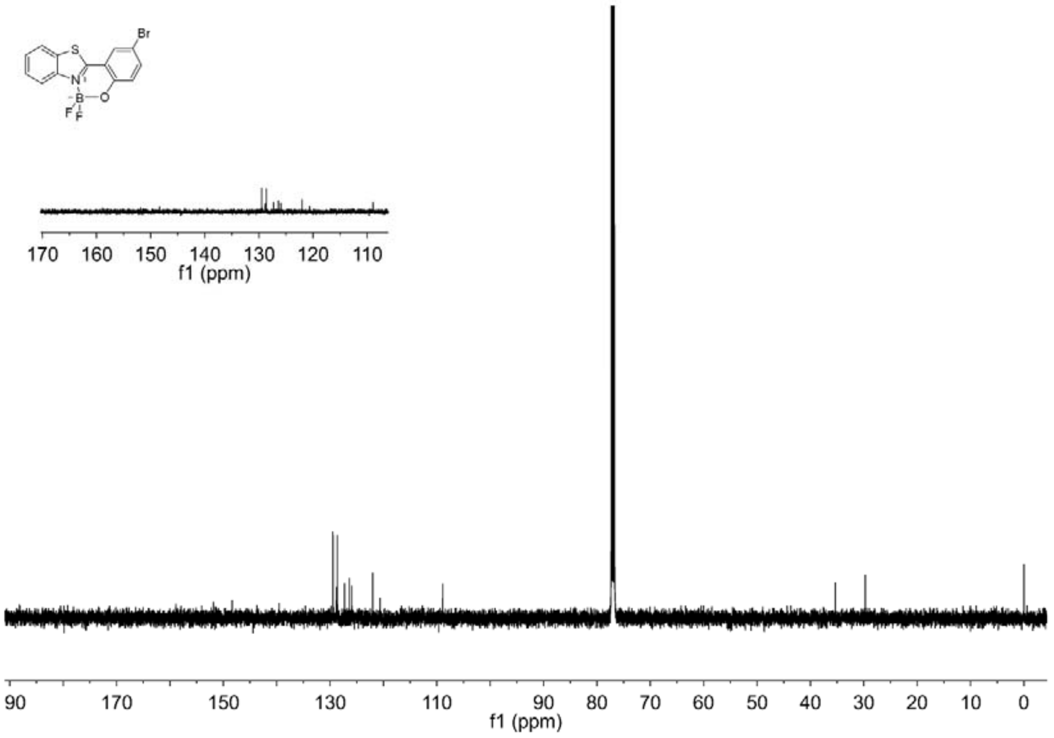
Br
S
N
B
O
F
F
170 160 150 140 130 120 110
f1 (ppm)
90 170 150 130 110 90 80 70 60 50 40 30 20 10 0
f1 (ppm)

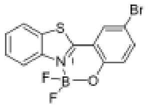

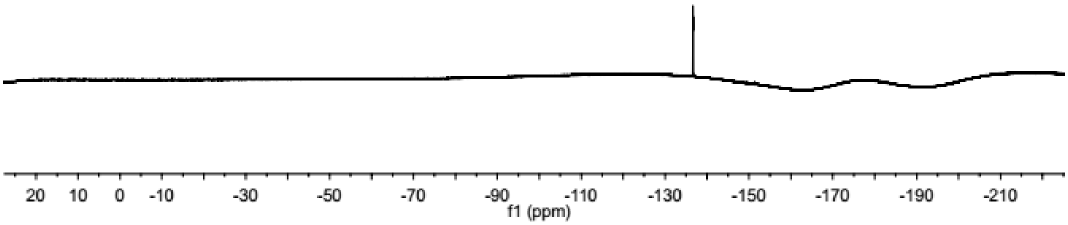

^{1}H NMR, ^{13}C NMR, ^{19}F NMR spectra of **7B** in chloroform-*d*.

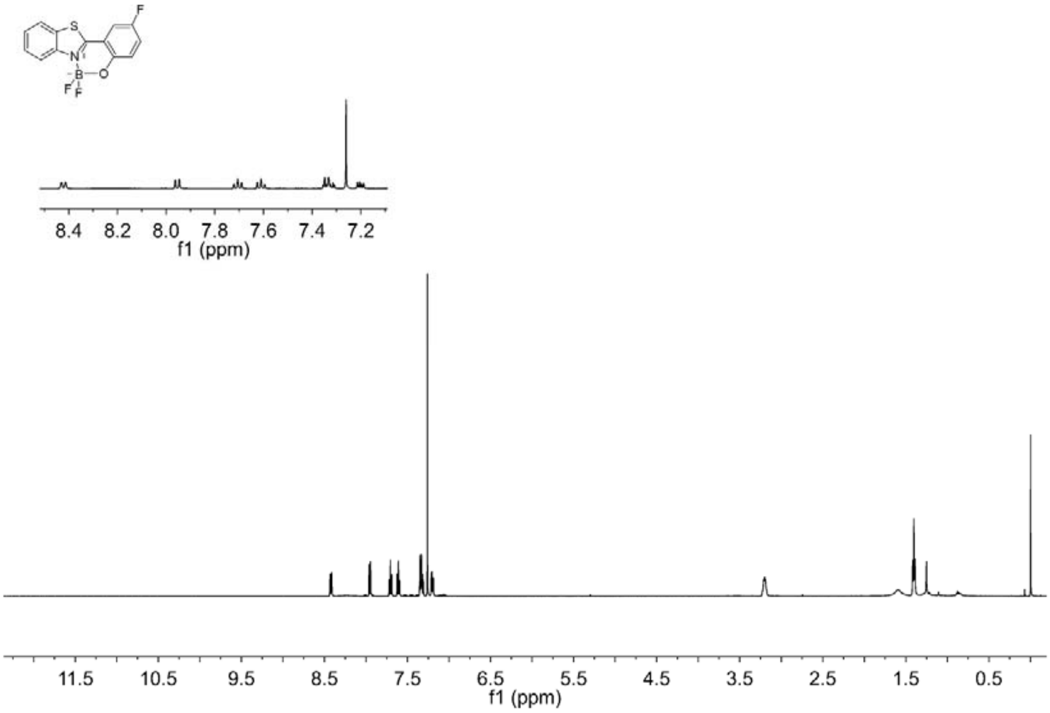
F
S
N
B
O
F F
8.4 8.2 8.0 7.8 7.6 7.4 7.2
f1 (ppm)
11.5 10.5 9.5 8.5 7.5 6.5 5.5 4.5 3.5 2.5 1.5 0.5
f1 (ppm)

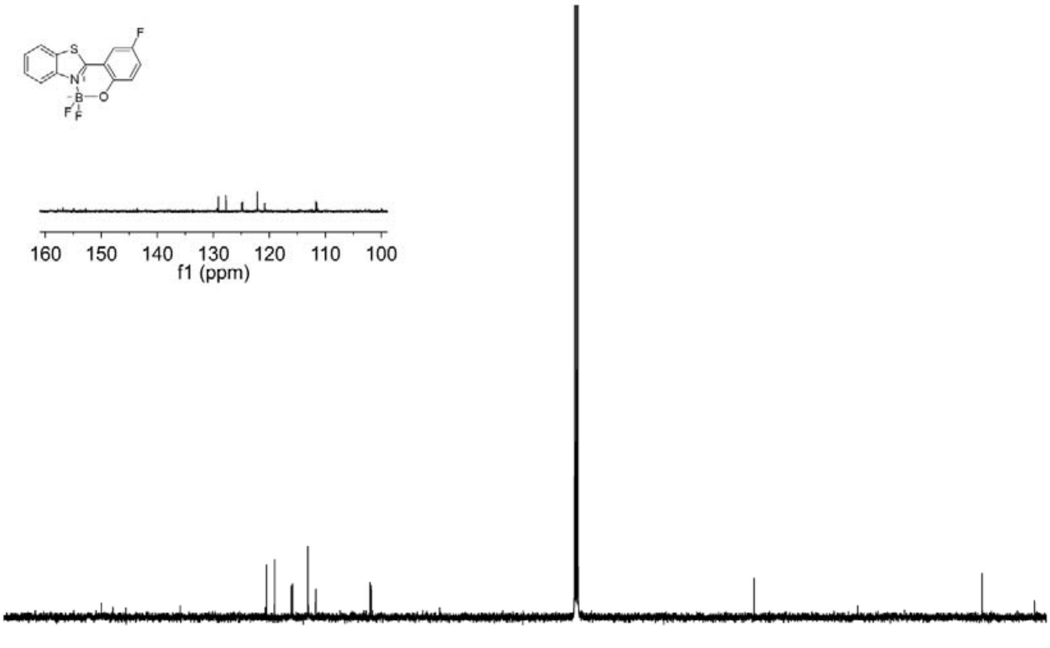
F
S
N
B
O
F F
160 150 140 130 120 110 100
f1 (ppm)
170 160 150 140 130 120 110 100 90 80 70 60 50 40 30 20 10 0
f1 (ppm)

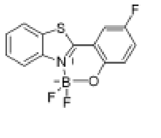

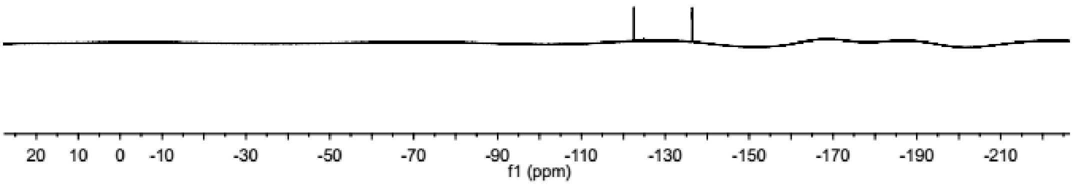

^{1}H NMR, ^{13}C NMR, ^{19}F NMR spectra of **8B** in chloroform-*d*.

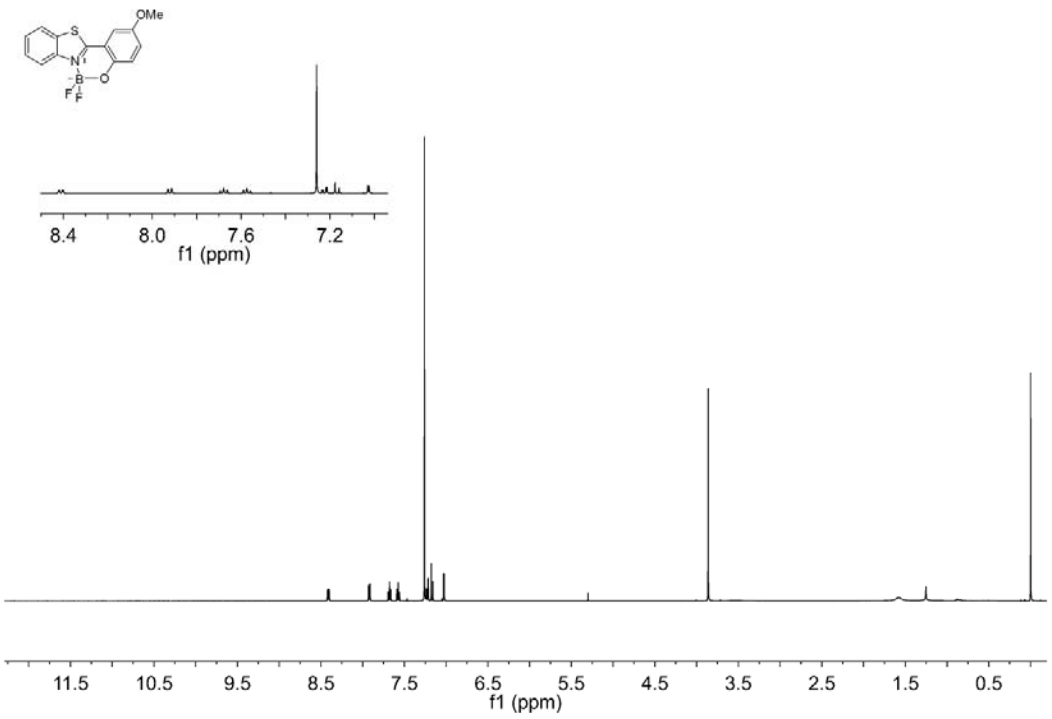
OMe
S
N
B
O
F
F
8.4
8.0
7.6
7.2
f1 (ppm)
11.5
10.5
9.5
8.5
7.5
6.5
5.5
4.5
3.5
2.5
1.5
0.5
f1 (ppm)

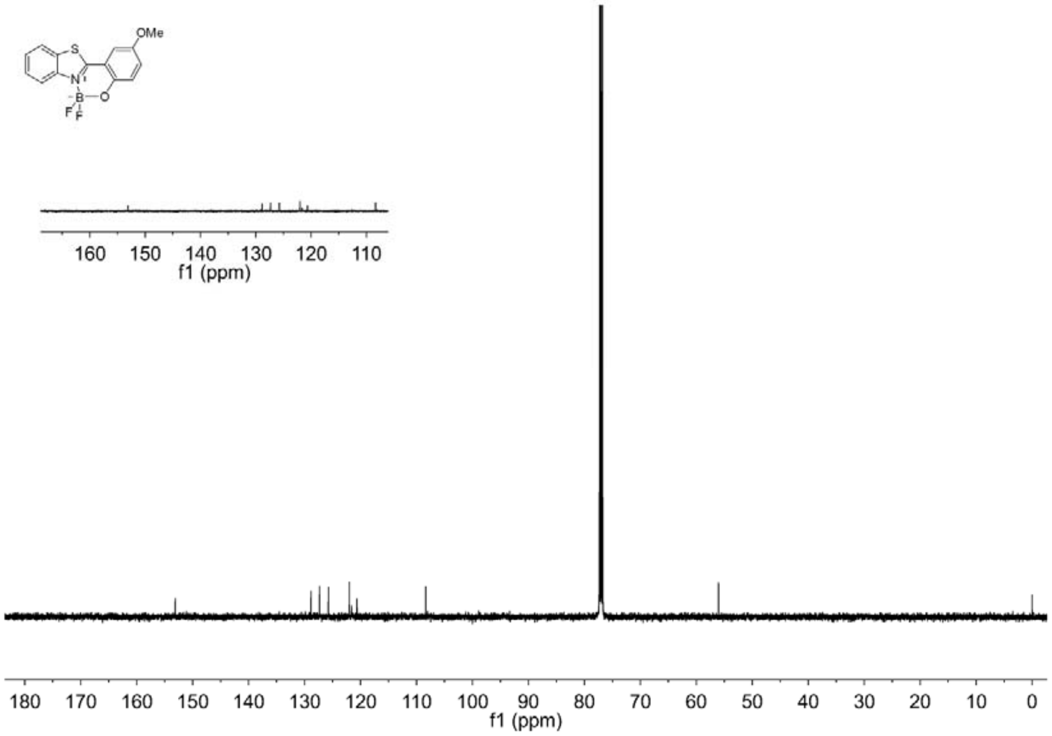
OMe
S
N
B
O
F
F
160
150
140
130
120
110
f1 (ppm)
180
170
160
150
140
130
120
110
100
90
80
70
60
50
40
30
20
10
0
f1 (ppm)

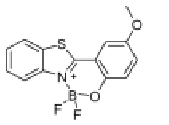

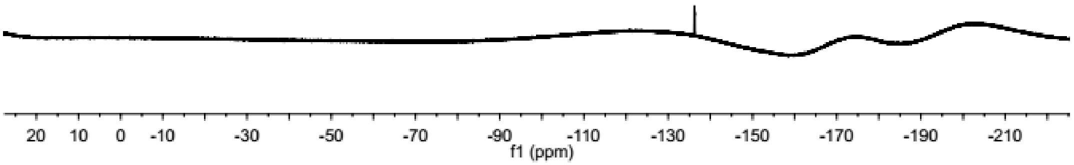

^{1}H NMR, ^{13}C NMR, ^{19}F NMR spectra of **9B** in chloroform-*d*.

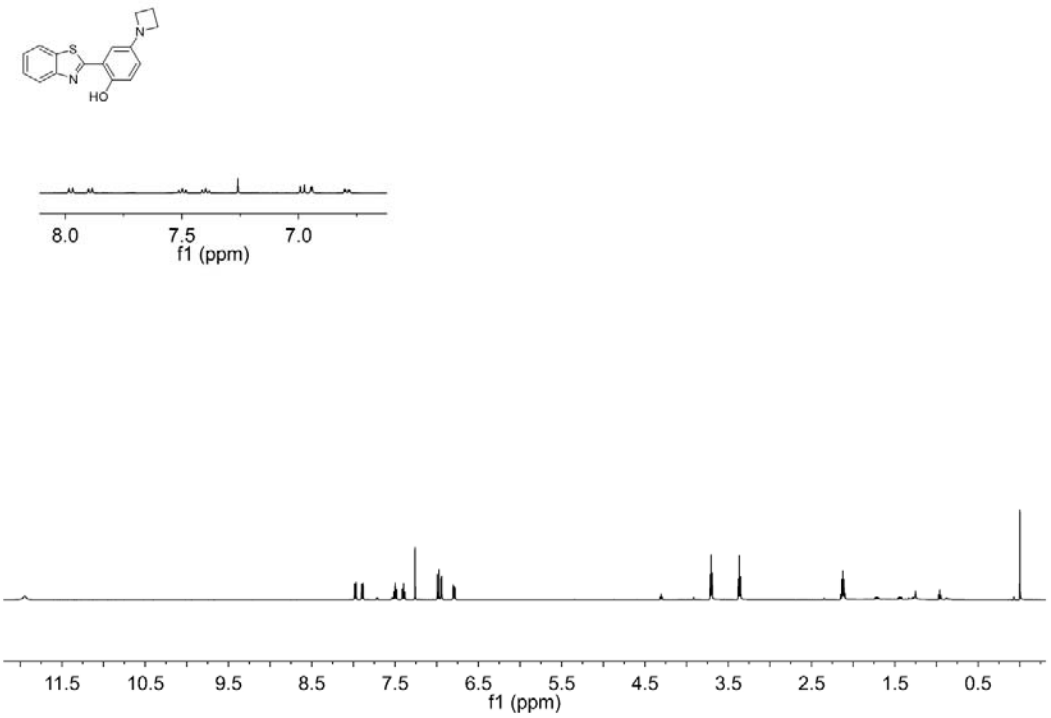

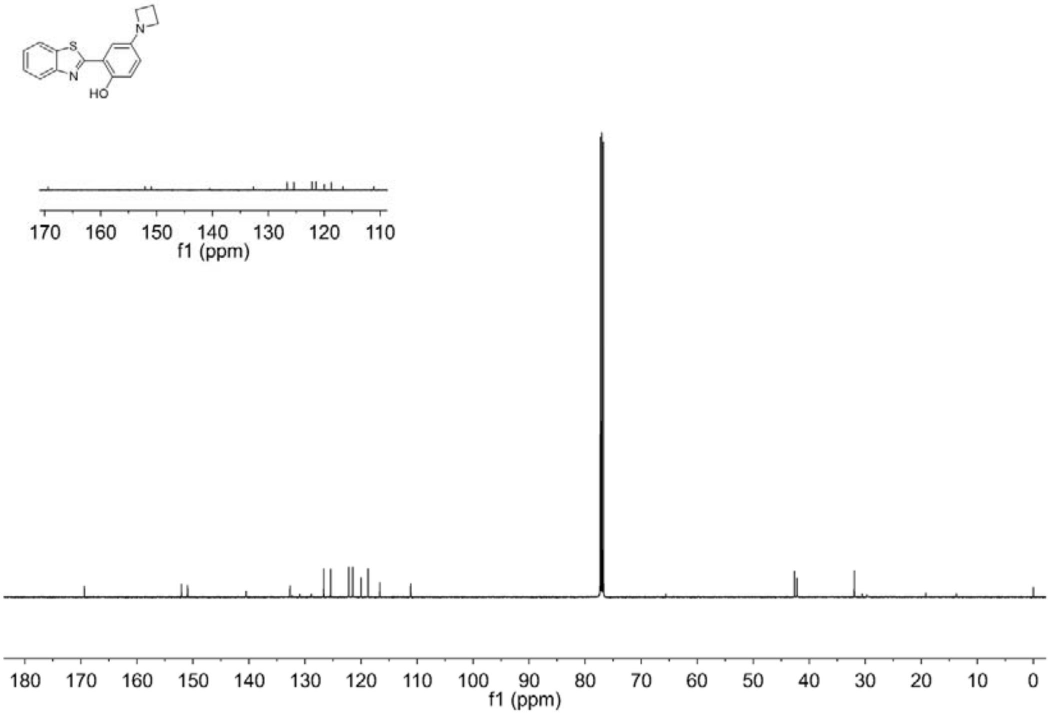

^{1}H NMR, ^{13}C NMR spectra of **11** in chloroform-*d*.

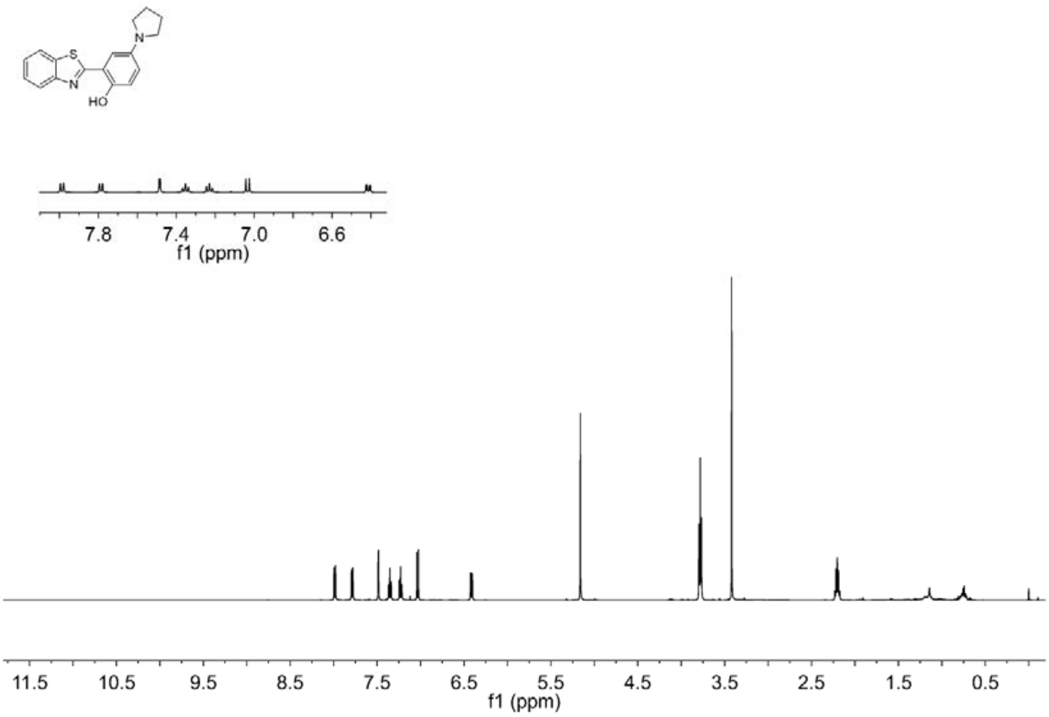

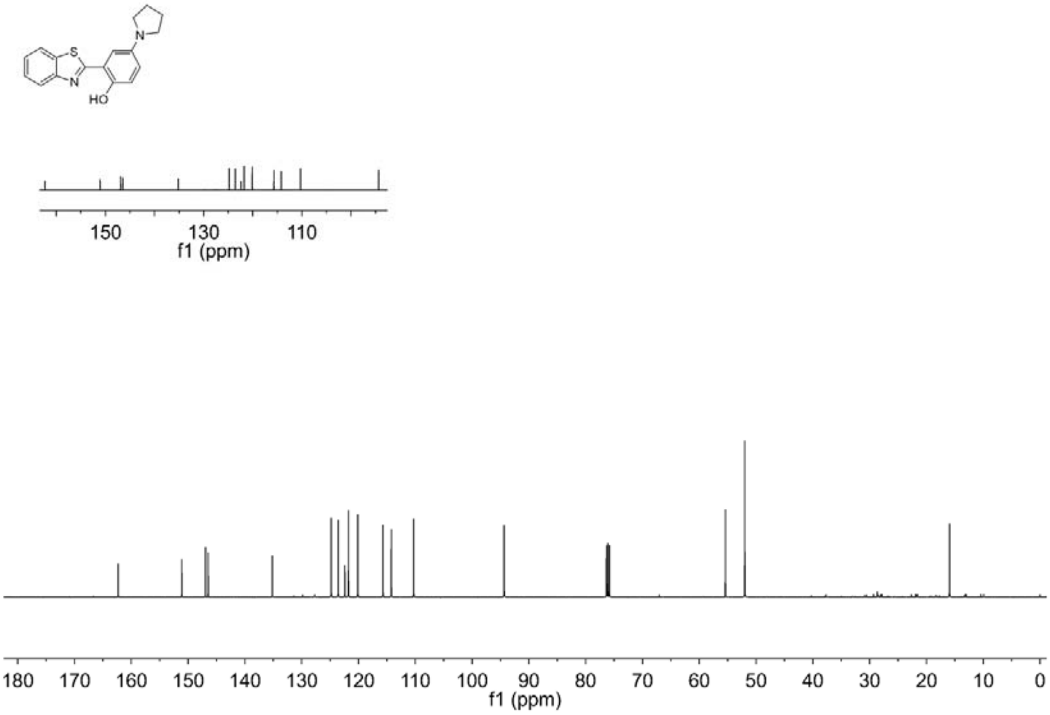

^{1}H NMR, ^{13}C NMR spectra of **12** in chloroform-*d*.

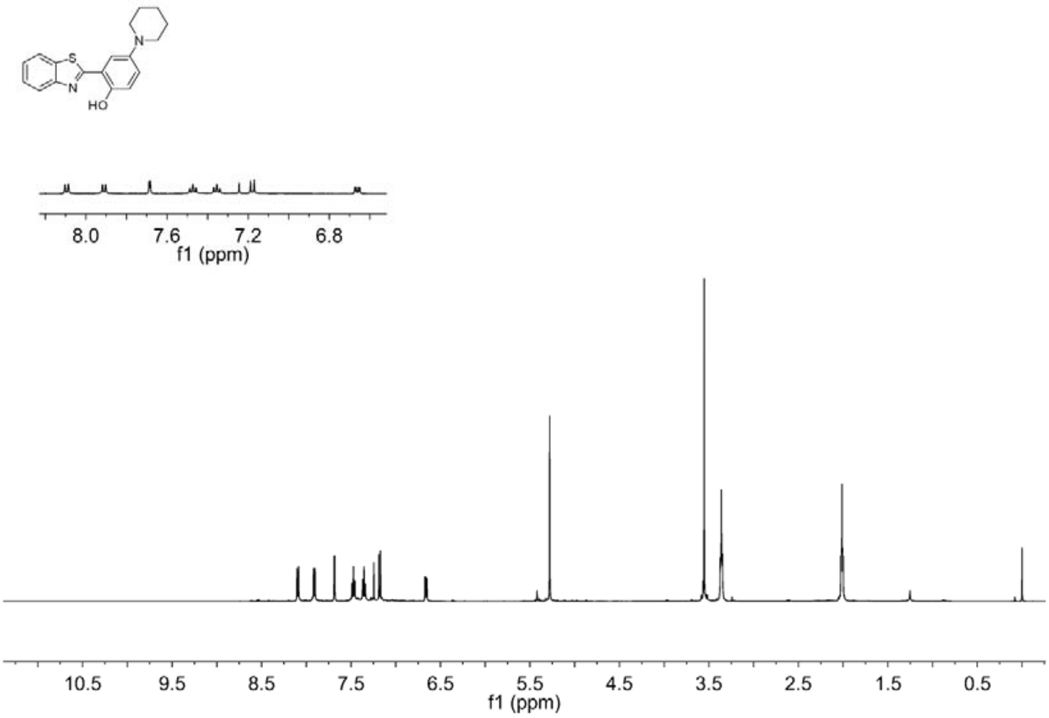

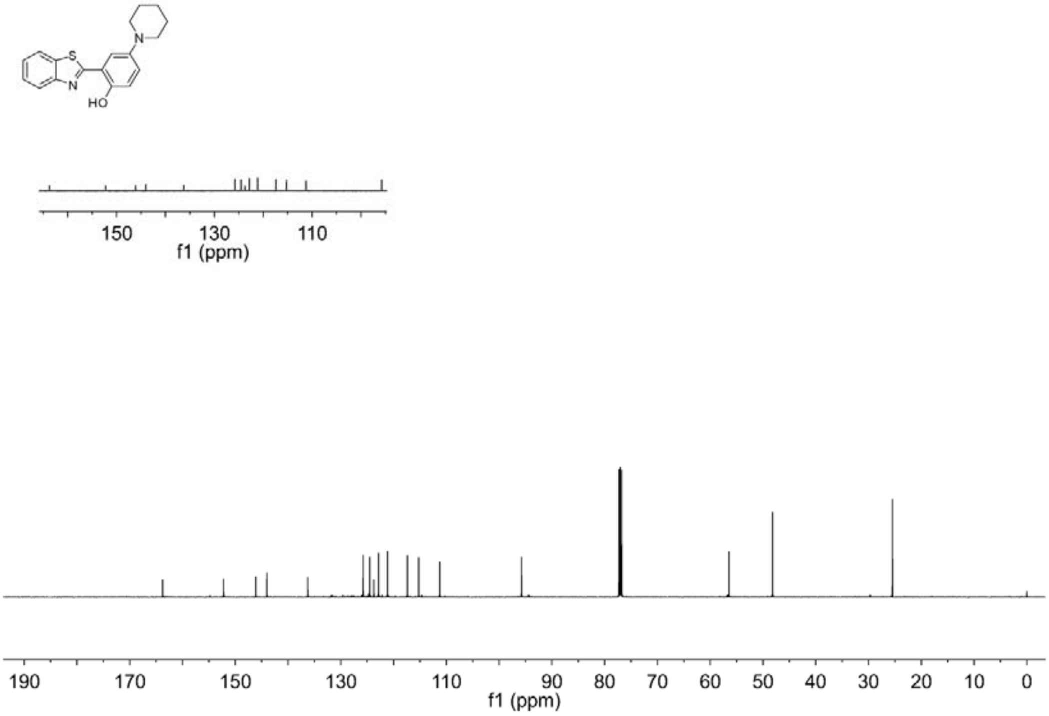

^{1}H NMR, ^{13}C NMR spectra of **13** in chloroform-*d*.

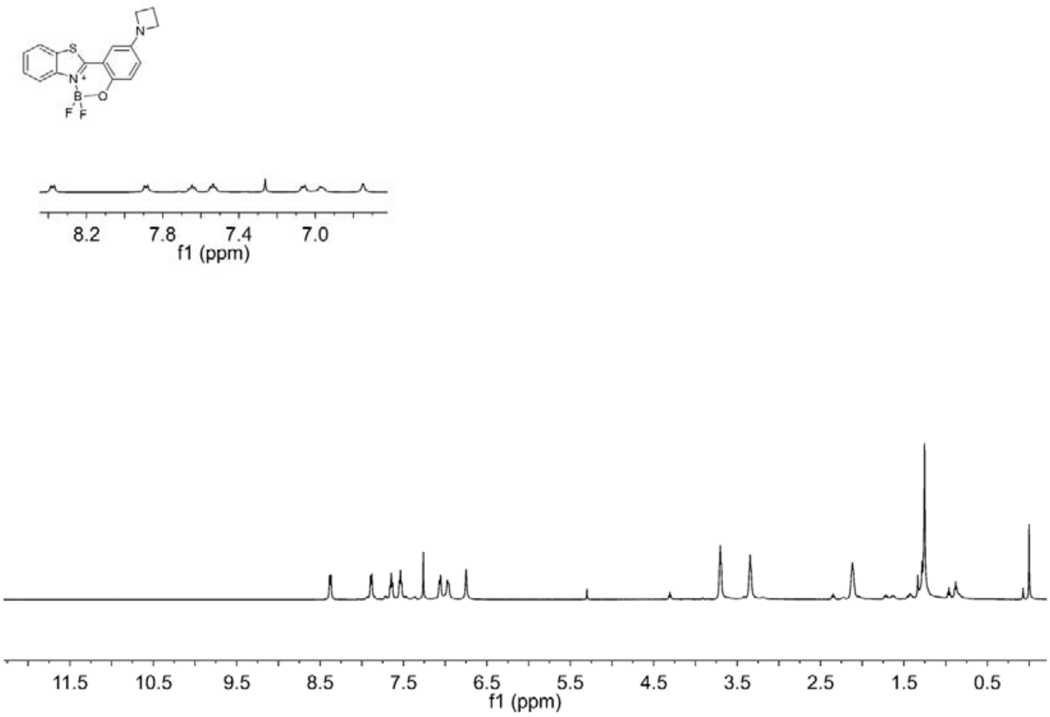
S
N
B
O
F F
8.2
7.8
7.4
7.0
f1 (ppm)
11.5
10.5
9.5
8.5
7.5
6.5
5.5
4.5
3.5
2.5
1.5
0.5
f1 (ppm)

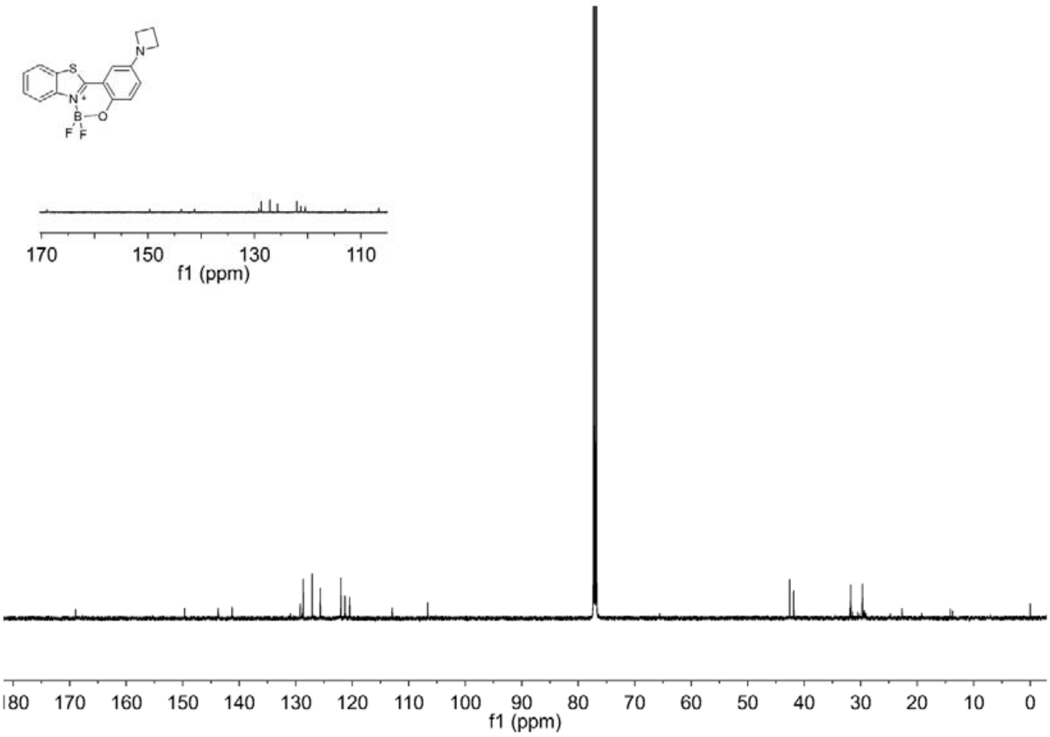
S
N
B
O
F F
170
150
130
110
f1 (ppm)
180
170
160
150
140
130
120
110
100
90
80
70
60
50
40
30
20
10
0
f1 (ppm)

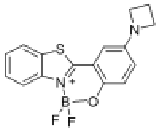

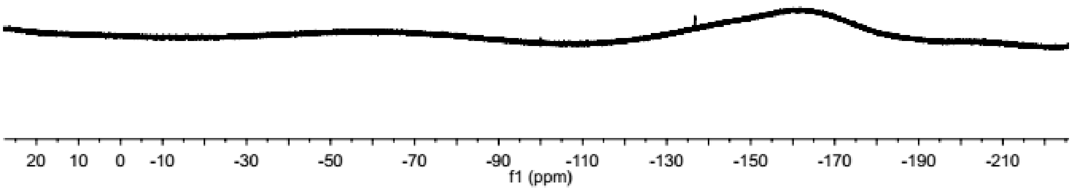

^{1}H NMR, ^{13}C NMR, ^{19}F NMR spectra of **11B** in chloroform-*d*.

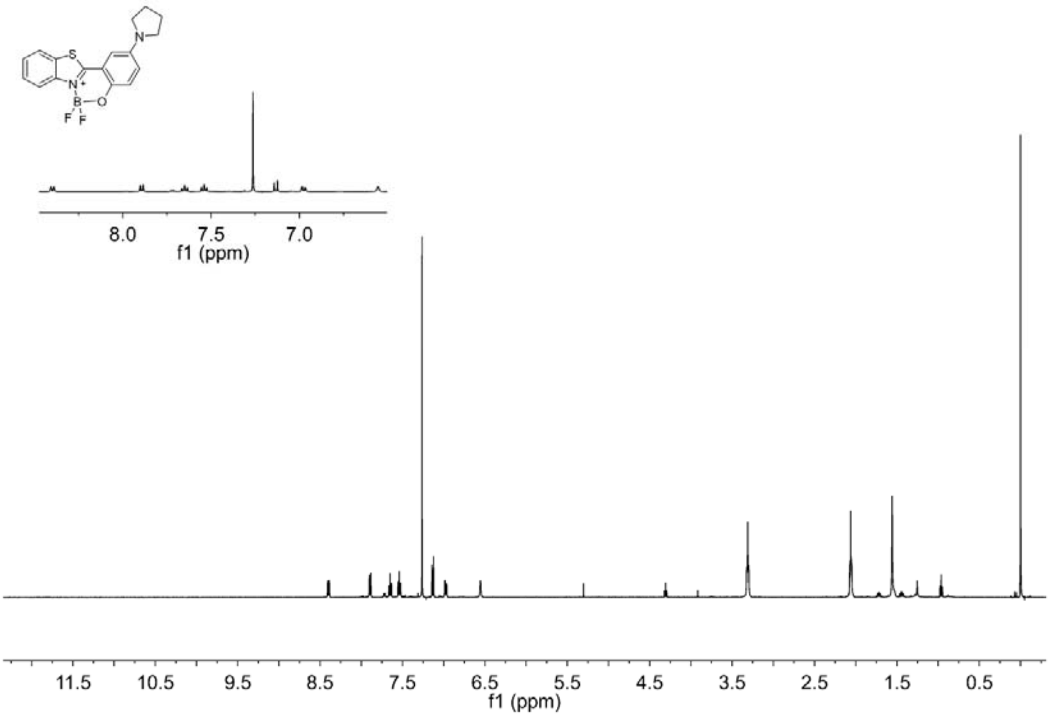

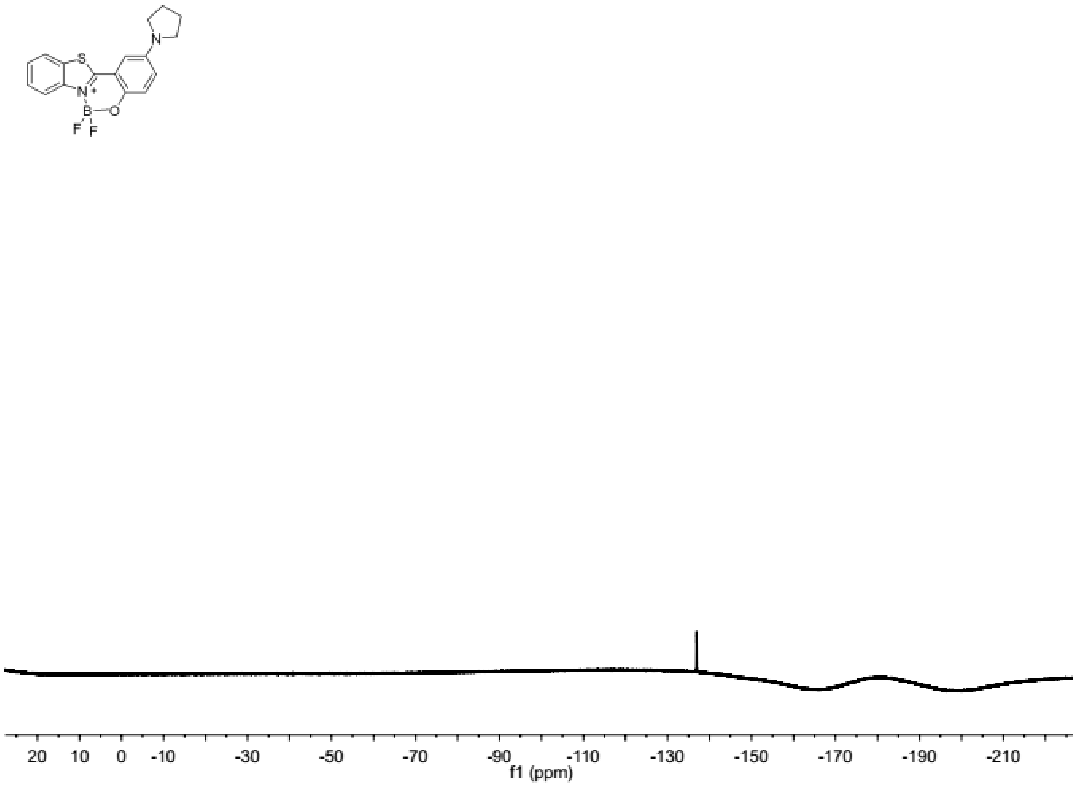

^{1}H NMR, ^{19}F NMR spectra of **12B** in chloroform-*d*.

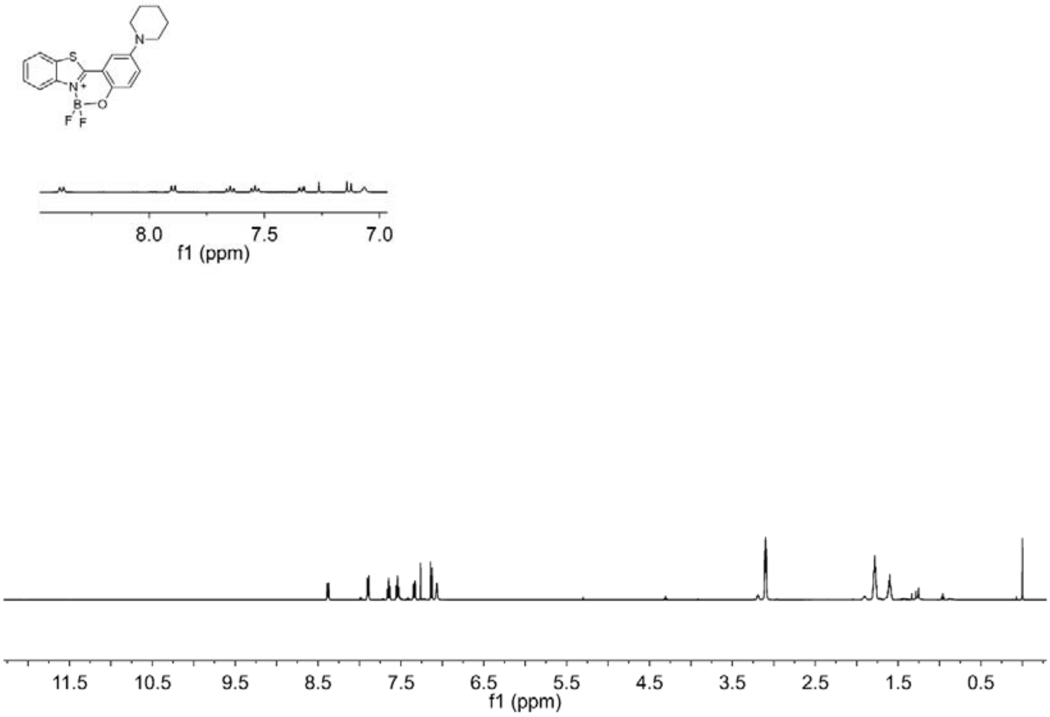
S
N+
B
O
F F
8.0 7.5 7.0
f1 (ppm)
11.5 10.5 9.5 8.5 7.5 6.5 5.5 4.5 3.5 2.5 1.5 0.5
f1 (ppm)

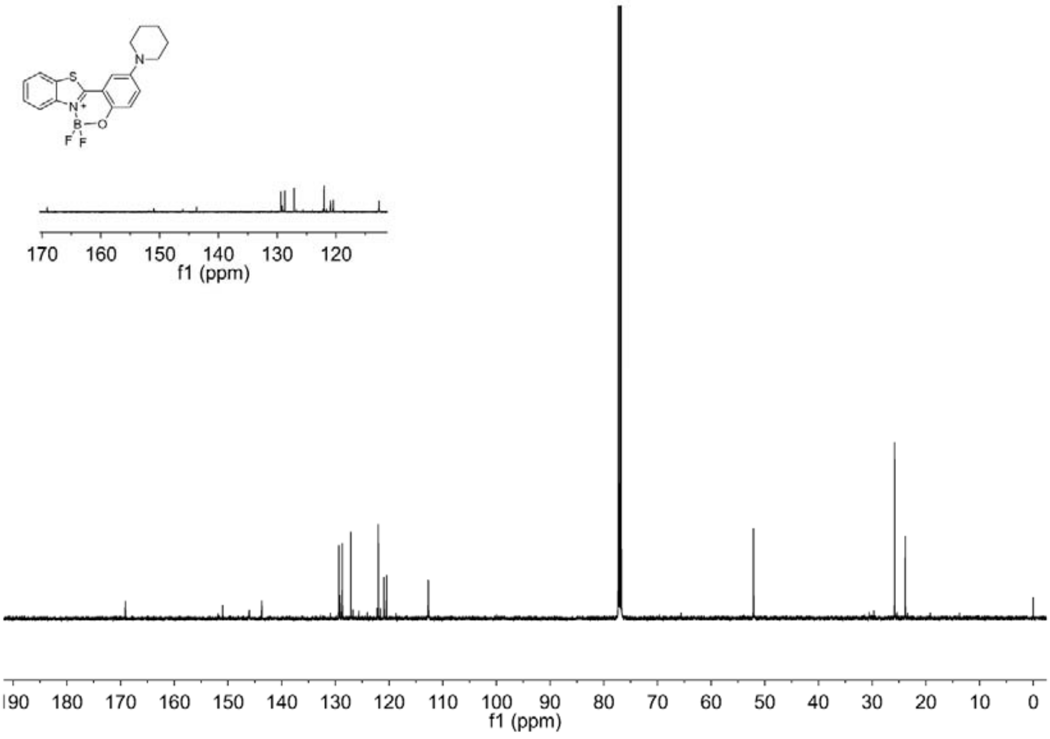
S
N+
B
O
F F
170 160 150 140 130 120
f1 (ppm)
190 180 170 160 150 140 130 120 110 100 90 80 70 60 50 40 30 20 10 0
f1 (ppm)

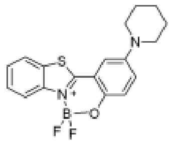

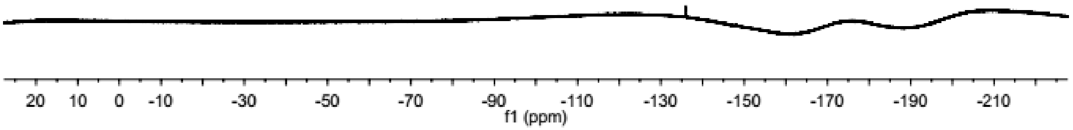

^{1}H NMR, ^{13}C NMR, ^{19}F NMR spectra of **13B** in chloroform-*d*.
